# Profiling of Chromatin Accessibility in Pigs across Multiple Tissues and Developmental Stages

**DOI:** 10.3390/ijms241311076

**Published:** 2023-07-04

**Authors:** Jingyi Bai, Yu Lin, Jiaman Zhang, Ziyu Chen, Yujie Wang, Mingzhou Li, Jing Li

**Affiliations:** Institute of Animal Genetics and Breeding, College of Animal Science and Technology, Sichuan Agricultural University, Chengdu 611130, China

**Keywords:** epigenomic profile, transcriptional regulation, newborn pigs, liver, postnatal development

## Abstract

The study of chromatin accessibility across tissues and developmental stages is essential for elucidating the transcriptional regulation of various phenotypes and biological processes. However, the chromatin accessibility profiles of multiple tissues in newborn pigs and across porcine liver development remain poorly investigated. Here, we used ATAC-seq and rRNA-depleted RNA-seq to profile open chromatin maps and transcriptional features of heart, kidney, liver, lung, skeletal muscle, and spleen in newborn pigs and porcine liver tissue in the suckling and adult stages, respectively. Specifically, by analyzing a union set of protein-coding genes (PCGs) and two types of transcripts (lncRNAs and TUCPs), we obtained a comprehensive annotation of consensus ATAC-seq peaks for each tissue and developmental stage. As expected, the PCGs with tissue-specific accessible promoters had active transcription and were relevant to tissue-specific functions. In addition, other non-coding tissue-specific peaks were involved in both physical activity and the morphogenesis of neonatal tissues. We also characterized stage-specific peaks and observed a close association between dynamic chromatin accessibility and hepatic function transition during liver postnatal development. Overall, this study expands our current understanding of epigenetic regulation in mammalian tissues and organ development, which can benefit both economic trait improvement and improve the biomedical usage of pigs.

## 1. Introduction

Pigs (*Sus scrofa*) are important agricultural animals of substantial economic value, serving as a major source of meat globally. In recent years, the pig has also emerged as an essential large animal model for biomedical research [1,2], providing multiple cells and tissues suitable for xenotransplantation and stem cell therapy in humans [3,4,5]. In-depth investigations of the genetic and epigenetic regulation of porcine tissues can provide theoretical guidance for the further improvement of economic traits and enhance biomedical potential.

Genomic DNA is tightly compacted in eukaryotic cells to ensure gene transcription control and DNA damage protection. In general, structurally loose chromatin (i.e., euchromatin) has more active gene transcription, while condensed chromatin (i.e., heterochromatin) confers transcriptional repression and genome stability [6]. For example, accessible cis-regulatory elements (e.g., promoters, enhancers, and silencers) located in open chromatins enable the binding of transcription factors (TFs) to activate or silence target genes [7]. This epigenetic control of gene expression is an important mechanism involved in the regulation of several biological processes (e.g., development) and diverse physical activities across different tissues [8,9,10,11,12].

The assay for transposase-accessible chromatin followed by sequencing (ATAC-seq) is a well-developed technique of high robustness that is widely used for measuring genome-wide chromatin accessibility and exploring epigenetic regulation [13,14]. A large number of studies have used ATAC-seq to characterize open chromatin accessibility across the tissues of embryonic or adult pigs [15,16,17,18,19] but not newborn pigs. The neonatal period of farm animals is of particular value in scientific research, as it represents the end of fetal development and the start of postnatal growth. Hence, understanding the molecular regulatory features in this stage may lay the foundation for the improvement of economic traits and phenotypes in adult animals. For example, low birth weight in pigs associated with genetic and environmental factors causes lifelong impairments in muscle development and growth [20,21].

The liver plays an essential, multifunctional role in mammals, including hematopoiesis in the embryonic and fetal stages and metabolism, immunity, and clotting in the postnatal stages [22,23,24,25,26,27,28,29]. In particular, the liver is the animal metabolic hub [29,30,31] and closely associated with economic traits (e.g., meat yield, feed efficiency, and growth rate) in pigs [32,33,34]. Moreover, porcine liver is a potential source of xenotransplantation in humans [35]. Accordingly, the analysis of chromatin accessibility dynamics and epigenetic regulation during porcine liver development is essential for understanding the molecular mechanisms of hepatic function transition throughout development and for improving xenotransplantation.

We thus analyzed chromatin accessibility and transcriptomic profiles in six tissues (heart, kidney, liver, lung, skeletal muscle (longissimus dorsi muscle), and spleen) of newborn (one-day-old) pigs and characterized dynamic chromatin accessibility and concomitant transcriptional changes during liver development. We identified tissue-specific and stage-specific accessible regions that regulated tissue-specific and hepatic function transition during development. Our study confirmed the transcriptional regulatory role of chromatin accessibility across promoters and other non-coding regions in tissue-specific physical activities and organ development and enriched the epigenetic profiling and genome regulation profiling of pigs across tissues and developmental stages. This information has implications for advanced breeding programs and biomedical applications. 

## 2. Results

### 2.1. Landscape of Chromatin Accessibility across Tissues and Postnatal Liver Development

**Figure 1:**
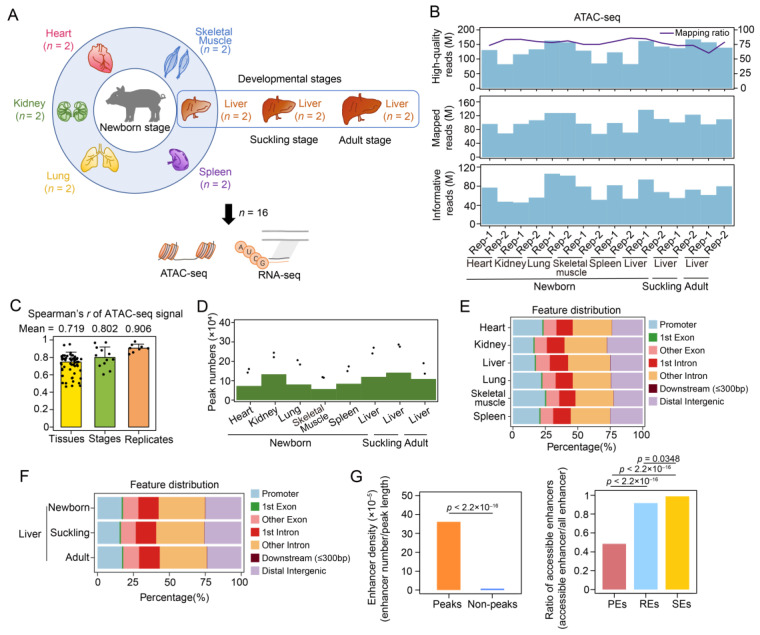
Basic features of the ATAC-seq data across tissue samples (*n* = 16). (**A**) Experimental design overview. The heart, kidney, liver, lung, skeletal muscle, and spleen tissue samples of newborn pigs and porcine liver samples obtained in the suckling and adult stages (each with two replicates) were collected for ATAC-seq and rRNA-depleted RNA-seq profiling, including the RNA-seq data of newborn tissue samples downloaded from the GSA. (**B**) High-quality read counts, mapping ratios (upper), mapped read counts (middle), and informative read counts (lower) are shown (see Materials and Methods). (**C**) Spearman’s correlation coefficients of the normalized ATAC-seq signals among 16 samples are shown in the following three categories: between tissues, between developmental stages of the liver, and between biological replicates. Data are shown as the mean ± SD. (**D**) The number of ATAC-seq peaks of merged replicates for each tissue in the newborn stage and each developmental stage of the liver are shown (green bars). The number of peaks identified in each replicate are indicated by dots. (**E**) Genomic distribution of consensus peaks for six tissues of newborn pigs. (**F**) Genomic distribution of consensus peaks for the livers of newborn, suckling, and adult pigs. (**G**) Comparison of enhancer density between accessible and inaccessible chromatins (left) and accessible enhancer ratios (defined as the proportion of accessible enhancers among enhancers with different activities identified in newborn porcine liver; right). These enhancers were identified in published research [40]. Enhancers with low, moderate, and high activity are shown as PEs (poised enhancers), REs (regular enhancers), and SEs (super enhancers).

To profile chromatin accessibility across multiple tissues in newborn pigs, we performed ATAC-seq to obtain genome-wide accessible chromatin regions (ACRs) in the heart, kidney, liver, lung, skeletal muscle, and spleen of animals aged one day old (newborn) (Figure 1A). We also examined chromatin accessibility changes during postnatal liver development by profiling the ACRs in porcine liver at 28 days (suckling) and 180 days (adult) using ATAC-seq (Figure 1A). We construct a total of sixteen libraries, generating 86.17–167.12 million (M) high-quality ATAC-seq reads with a mapping ratio of 60.18–85.88% for each sample (Figure 1B and Table S1). After filtering and deduplication, we obtained 45.83–106.01 M informative reads (Figure 1B and Table S1). The fragment size distribution showed decreasing and periodic peaks corresponding to nucleosome-free (<100 bp) and mono- (~200 bp), di- (~400 bp), and tri-nucleosomal (~600 bp) fragments [9] (Figure S1). The nucleosome-free regions which can be captured via ATAC-seq at the 5′ ends of genes are closely associated with transcription initiation [36]. As expected, the normalized ATAC-seq signals were enriched in the transcription start site (TSS) region (i.e., 2 kb upstream and 500 bp downstream of TSS) in all the samples (Figure S2). To further observe other epigenetic signatures in the promoters, we downloaded the previous epigenetic data, including H3K4me3 and H3K27me3 (which are associated with the activation and repression of promoters, respectively) [37]. The active histone mark H3K4me3 was enriched in the promoter regions, a finding which was similar to that for the ATAC-seq signals, whereas H3K27me3 was depleted (Figure S3). Correlation analysis of the 16 samples not only demonstrated the high reproducibility of the ATAC-seq data but also confirmed divergent chromatin accessibility patterns among multiple tissues (Figure 1C and Figure S4A). The correlation of chromatin accessibility between newborn and suckling livers (Spearman’s *R* = 0.953, *p* < 2.2 × 10^–16^) was higher than that between newborn and adult livers (Spearman’s *R* = 0.831, *p* < 2.2 × 10^–16^), which indicated global similarity in chromatin structure during the early postnatal development of the liver.

We identified 115,523–270,293 ATAC-seq peaks in each replicate of six tissues and 136,452–286,446 peaks in each liver sample in the three stages using MACS2 (Figure 1D and Table S2). To acquire representative ACRs for each tissue or stage, the peaks of replicates were merged, allowing us to obtain 57,126–133,254 and 109,003–141,689 consensus peaks for each tissue from newborn pigs and for each developmental stage of the liver, respectively (Figure 1D and Table S2). These peaks were used for subsequent analyses. In all the tissues, peak annotation showed that the majority of merged peaks intersected with intronic (41.80–46.43%, mean = 43.93%) and intergenic regions (22.49–27.59%, mean = 24.76%). To some extent, these intronic and intergenic regions might also harbor active cis-regulatory elements, such as enhancers, insulators, or silencers [38,39]. The remaining peaks were located in the promoter (15.77–24.80%, mean = 20.36%) and exonic (10.38–11.62%, mean = 10.92%) regions (Figure 1E). Peaks identified in the liver samples across developmental stages showed similar genomic distributions (Figure 1F). 

Additionally, we used enhancer data from liver samples in the newborn stage published in [40] and observed a significant enrichment of enhancers, especially highly active enhancers, in accessible chromatin regions. These observations confirmed that non-coding accessible regions usually contain substantial cis-regulatory elements to implement transcriptional regulation [41] (Figure 1G).

### 2.2. Coordination between Gene Transcription and Chromatin Accessibility

**Figure 2:**
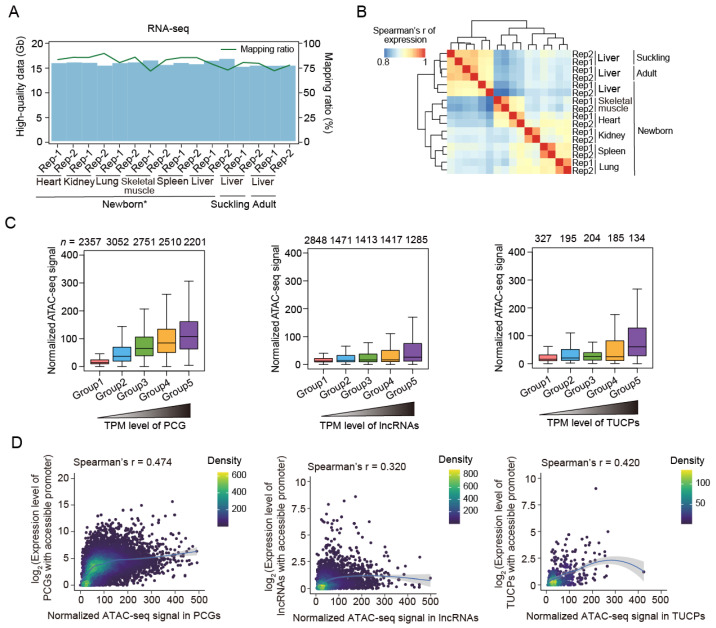
RNA-seq data summary and the close association between transcriptional activity and chromatin accessibility. (**A**) Volume (blue bars) and mapping ratios (green lines) of high-quality RNA-seq data. Downloaded data of newborn tissue samples are labeled with ‘*’. (**B**) Spearman’s correlation heatmap of transcriptional profiles (TPM) among the 16 samples, showing a tissue-dominant pattern. (**C**) Normalized ATAC-seq signals of the PCGs, lncRNAs, and TUCPs increased with the expression level. Genes were classified into five groups, with expression increasing from groups 1 to 5. Gene numbers are indicated above the plots. The data of the newborn skeletal muscle samples are shown here. (**D**) The correlation between chromatin accessibility (measured via normalized ATAC-seq signal) and gene expression (measured via TPM using RNA-seq data) in the skeletal muscle tissue in the newborn stage. The gray dashed lines represent the fitting lines.

Chromatin accessibility is usually qualitatively and quantitatively associated with gene transcription. To decipher gene transcriptional activity regulated by ACRs in pig somatic tissues and during liver development, we built transcriptional profiles for the same samples (*n* = 16) using both the downloaded and newly generated rRNA-depleted RNA-seq data (15.91 Gb of high-quality data per sample; Figure 2A and Table S3). As expected, the RNA-seq data exhibited a tissue-dominant pattern and good reproducibility between biological replicates (Figure 2B and Figure S4B), demonstrating high reliability. Around two-thirds of the protein-coding genes (PCGs, 60.67–67.42%, transcripts per million (TPM) > 0.5), 48.30–63.54% of the long noncoding RNAs (lncRNAs, TPM > 0.1), and 45.40–65.53% of the transcripts of unknown coding potential (TUCPs, TPM > 0.1) had detectable expression in each tissue or developmental stage (Figure S5). 

Based on the transcriptional data, we observed that the genes with accessible promoter regions had significantly upregulated expression compared to those without open promoters across tissues and developmental stages (Figure S6). In addition, genes with higher expression exhibited higher levels of chromatin accessibility (Figure 2C and Figure S7). A moderate correlation (Spearman’s *R* = 0.303–0.525) between the ATAC-seq signal and gene expression, as measured via RNA-seq, confirmed the important role of open chromatin in active gene transcription (Figure 2D and Figure S8).

### 2.3. Differences in Chromatin Accessibility between Tissues

**Figure 3:**
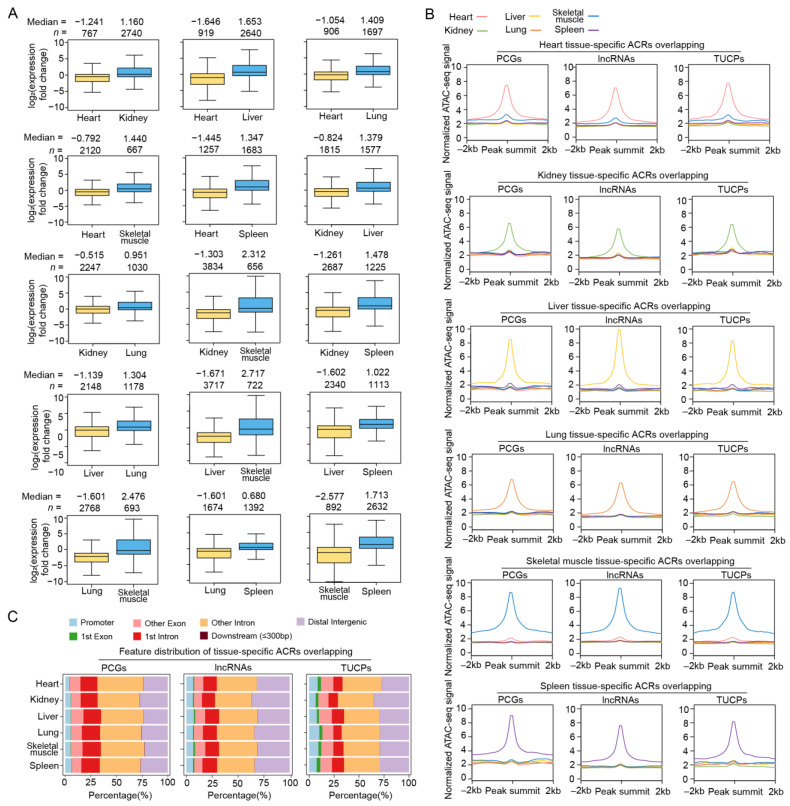
Basic characteristics of differential ACRs between tissues. (**A**) Comparison of expression level for genes whose promoter regions are located in differential ACRs between tissues. Gene numbers and median values are shown above the plots. (**B**) Normalized ATAC-seq signals of tissue-specific ACRs in 4 kb windows centered on the peak. The lines represent the average values for different tissues. (**C**) Genomic distribution of tissue-specific ATAC-seq peaks relative to the genic features of PCGs (left), lncRNAs (middle) and TUCPs (right).

We next sought to explore the differences in chromatin accessibility between tissues and transcriptional changes potentially caused by these differential ACRs. We observed that the differential peaks between tissues showed corresponding biases in gene transcription (Figure 3A). In total, we observed 5565–17,011 tissue-specific peaks in the PCG promoter regions of each tissue (Figure 3B and Table S2). The kidney-specific peaks overlapped with the promoter regions of 970 PCGs, followed by liver- and spleen-specific peaks, which overlapped with 824 and 574 PCG promoters, respectively. In addition, the heart- and muscle-specific peaks intersected with the least number of PCG promoter regions (*n* = 220 and 270, respectively) (Table S2). Interestingly, compared to all the ACRs identified in each tissue, tissue-specific peaks were located less in the promoter regions (averagely 20.36% vs. 5.21%) and more in the intronic regions (averagely 43.93% vs. 58.95%) (Figure 3C), suggesting that cis-regulatory elements inhabiting the intergenic and intronic regions, such as enhancers and insulators, are probably more important than promoters for tissue-specific functions. 

### 2.4. Functional Implications of Tissue-Specific Peaks

**Figure 4:**
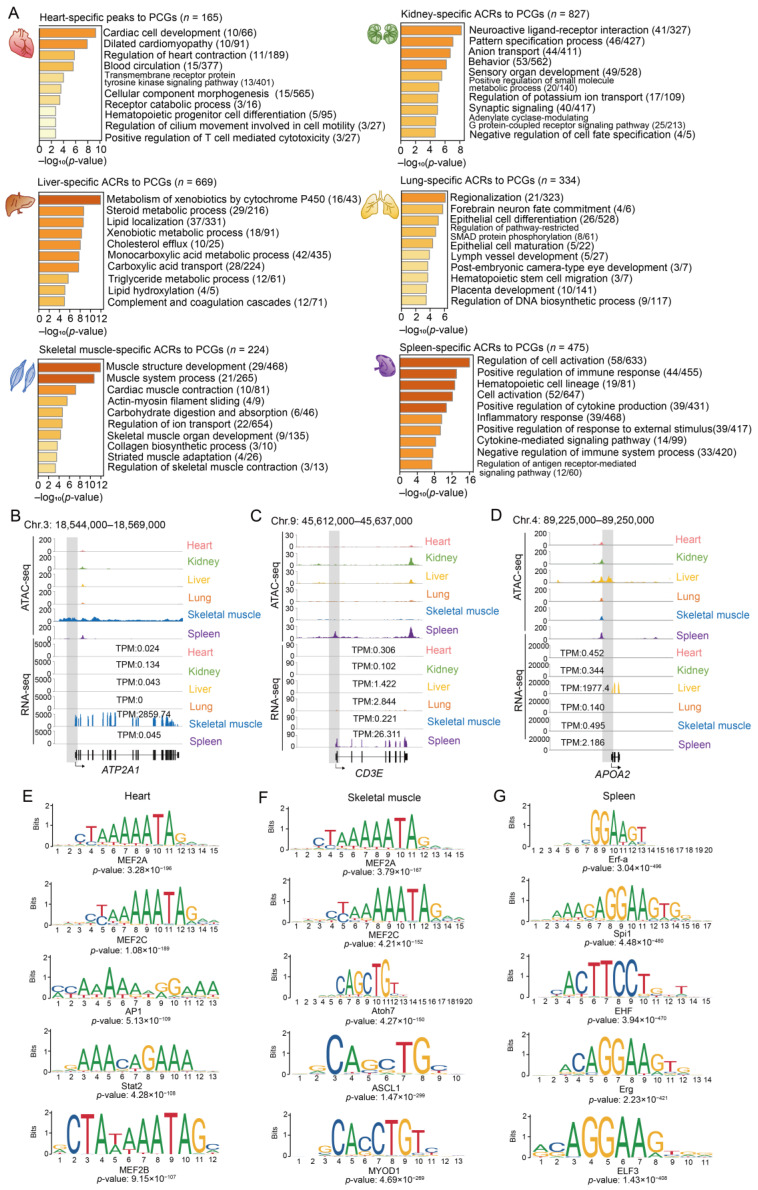
Functional and TF enrichment of tissue-specific ACRs. (**A**) Top ten significantly enriched Gene Ontology-Biological Process (GO-BP) terms or Kyoto Encyclopedia of Genes and Genomes (KEGG) pathways for PCGs with promoter regions overlapping tissue-specific peaks in the heart, kidney, liver, lung, skeletal muscle, and spleen. (**B**–**D**) Chromatin accessibility and gene expression levels in skeletal-muscle-specific ACRs overlapping the promoter regions of tissue markers, including *ATP2A1* (essential for removing calcium from the cytoplasm in the skeletal muscle, which is required for muscle relaxation [48,49,50,51]; (**B**) *CD3E* (encoding CD3-epsilon polypeptide and participating in T-cell development [52]; and (**C**) *APOA2* (playing a role in fatty acid and glucose metabolism [53,54]; (**D**). The grey squares indicate the promoter regions of the corresponding genes. (**E**–**G**) The top five enriched TF-binding motifs identified in the heart-, skeletal-muscle- and spleen-specific ACRs using the JASPAR database in the newborn stage.

Functional enrichment analysis showed PCGs with promoter regions overlapping tissue-specific ACRs, despite their small number and short length, were significantly enriched for the biological processes associated with each tissue (Figure 4A). For example, PCGs with heart-specific accessible promoters were mainly involved in cardiac cell development (e.g., *MYH6* and *TBX18*) and blood circulation (e.g., *ADCY6* and *MYBPC3*) (Figure S9A–D); PCGs with kidney-specific open promoters were enriched for terms related to the maintenance of pH balance and electrolyte homeostasis (such as the ‘regulation of potassium ion transport’ and ‘anion transport’,), excretion of harmful metabolites (‘positive regulation of small molecule metabolic process’), and early nephron formation (‘pattern specification process’, ‘negative regulation of cell fate specification’); PCGs with lung-specific promoters were overrepresented by those participating in epithelial cell differentiation, such as *BMPR2* and *NKX2-1* (Figure S9E,F); PCGs with promoters in skeletal-muscle-specific ACRs were enriched for ‘muscle contraction’, including *SCN4A* and *ATP2A1* (Figure 4B and Figure S9G); PCGs with promoters exclusively accessible in the spleen, a lymphoid organ playing important roles in immune response, were most relevant for immune processes, such as the ‘positive regulation of immune response’ (including *ADA* and *CD3E*) and ‘inflammatory response’ (including *HCK* and *VCAM1*) (Figure 4C and Figure S9H–J); and liver-specific accessible PCGs were mainly associated with metabolic processes, such as ‘lipid localization’ (e.g., *APOA2*, *APOB* and *APOH*), the ‘triglyceride metabolic process (e.g., *APOA2*, *MOGAT1* and *SOAT2*), and the ‘xenobiotic metabolic process’ (e.g., *SULT2A1* and *NR1I2*) (Figure 4D and Figure S9K–P).

To understand whether the vast majority (>80%) of tissue-specific peaks in the intronic and intergenic regions play a role in somatic tissue function, we annotated these peaks using GREAT. Our results showed that these peaks were relevant to specific tissue functions, as well as morphogenesis and differentiation in newborn tissues, especially the heart and skeletal muscle (Figure S10). This suggests that cis-regulatory elements, such as enhancers and silencers, probably participate more than promoters in the transcriptional regulation of developmental and morphogenic processes. 

To further explain which TFs are involved in transcriptional regulation via chromatin accessibility, we identified the enriched TFs in tissue-specific ACRs using motif analysis. We found that the majority of the TFs were relevant to tissue-dependent functions, including MEF2A (maintaining cyto-architectural integrity [42]) and MEF2D (regulating the cell cycle of neonatal cardiomyocytes [43]), which are enriched in heart-specific ACRs and responsible for maintaining normal heart function; MEF2C [44] and MYOD1 [45], which are enriched in skeletal-muscle-specific ACRs and regulate skeletal muscle regeneration and formation; and Spi1 [46] and IKZF1 [47], which were identified in the spleen-specific ACRs and participate in the generation of lymphoid lineages (Figure 4E–G and Figure S11, Table S4).

These results highlight the important regulatory roles of tissue-specific ACRs in the functional implementation of corresponding tissues, by both controlling the PCG expression and accessibility of non-coding regions, in which TFs probably play important roles.

### 2.5. Dynamic ACRs during Liver Development

**Figure 5:**
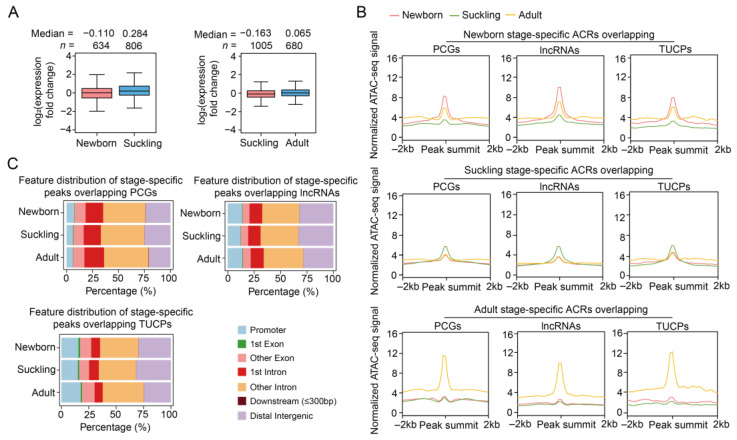
Basic characteristics of dynamic ACRs during postanal liver development. (**A**) Comparison of the expression levels for genes whose promoter regions overlap differential ACRs between neighboring stages. Gene numbers and median values are shown above the plots. (**B**) Normalized ATAC-seq signals of stage-specific ACRs in 4 kb windows centered on the peak. The lines represent average values in different developmental stages. (**C**) Genomic distribution of stage-specific ATAC-seq peaks relative to the genic features of the PCGs (upper left), lncRNAs (upper right), and TUCPs (lower).

To profile dynamic chromatin accessibility during postnatal liver development, we first identified 14,416–28,053 differential ATAC-seq peaks between neighboring stages, which showed consistent expression biases between stages for PCGs with promoters overlapping these peaks (Figure 5A). These observations highlight the functional significance of dynamic ACRs during liver development. 

Next, stage-specific ACRs were obtained, with a total of 3.09–4.85 Mb overlapping PCGs, 2.79–4.48 Mb intersecting lncRNAs, and 0.28–0.62 Mb overlapping TUCPs across the three stages. These included newborn-, suckling-, and adult-specific ACRs overlapping the promoter regions of 731, 1028, and 612 PCGs, respectively (Figure 5B and Table S2). The genomic distribution of the stage-specific ATAC-seq peaks was similar to that of the tissue-specific peaks, i.e., more peaks were located in intronic regions (59.27% vs. 47.09%) compared to the peaks identified in each stage (Figure 5C). The TFs enriched in these stage-specific ACRs were also identified, as shown in Figure S12 and Table S5.

### 2.6. Biological Meaning of Stage-Specific ACRs during Liver Development

**Figure 6:**
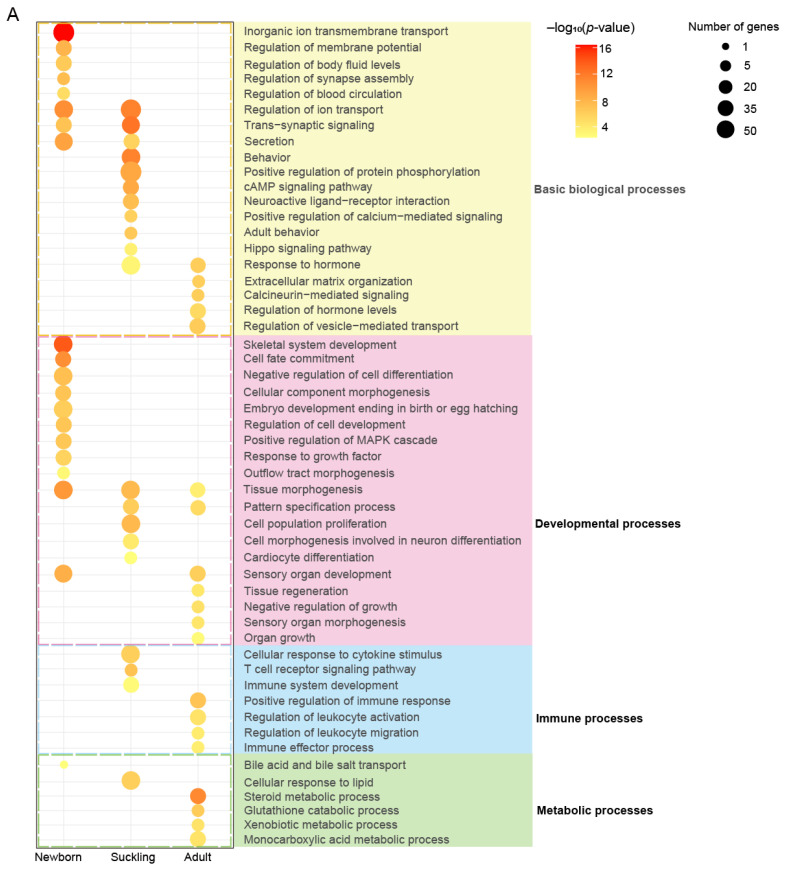

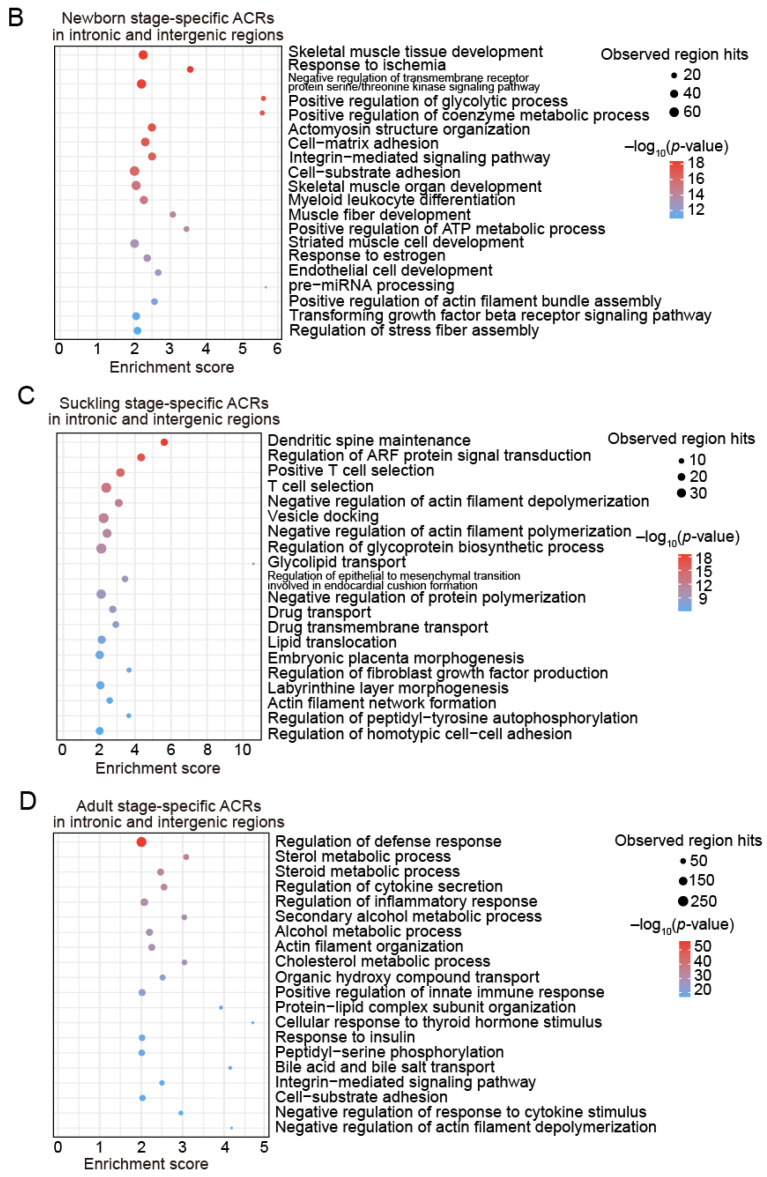
Functional enrichment analyses of stage-specific ACRs during postnatal liver development. (**A**) The top 20 significantly enriched GO-BP terms and KEGG pathways for PCGs with promoter regions located in stage-specific ACRs. The colorful shades of the terms highlight the term categories, including general biological processes (yellow), developmental processes (pink), immune processes (blue), and metabolic processes (green). (**B**–**D**) Bubble plots showing the enrichment of GO-BP terms in the newborn (**B**), suckling (**C**), and adult (**D**) stage-specific ACRs in intronic and intergenic regions. The enrichment analyses were performed using the GREAT software (v 4.0.4).

To infer the functional implications of dynamic ACRs during postnatal liver development, we performed functional enrichment analyses for the PCGs with promoters overlapping stage-specific ACRs, as well as the other non-coding stage-specific ACRs. Both enrichment analyses demonstrated that the ACRs are closely associated with functional liver features in different developmental stages, from hematopoiesis and tissue development in the newborn stage to the immune and metabolic functions in adults (Figure 6A). 

In addition to the general biological processes associated with signaling and ion transport, the PCGs with accessible promoters in the newborn stage showed the strongest enrichment in developmental processes (e.g., ‘embryo development ending in birth or egg hatching’, ‘cell fate commitment’, and the ‘regulation of cell development’) compared to other stages. These genes were also exclusively enriched for the ‘regulation of blood circulation’, corresponding to the hepatic function of hematopoiesis and regulating circulating blood volume. In addition, the PCGs with open promoters in the suckling or adult stages were related to immunity (e.g., ‘cellular response to cytokine stimulus’, ‘T cell receptor signaling pathway’, and ‘immune system development’) and metabolism (e.g., ‘steroid metabolic process’, ‘xenobiotic metabolic process’, and ‘monocarboxylic acid metabolic process’), respectively. These results support the hypothesis that the developing liver in the suckling stage has high immune activity, while the mature liver represents the metabolic hub of lipids, glucose, and xenobiotics.

The enrichment analysis of the stage-specific ACRs located in intronic and intergenic regions using GREAT showed similar results, supporting the notion that changing chromatin accessibility has regulatory roles in functional transition during liver development, probably via cis-regulatory elements, such as enhancers enriched in these ACRs (Figure 1G). The intronic and intergenic regions specifically accessible in the newborn stage were exclusively enriched for terms related to hematopoiesis (including ‘myeloid leukocyte differentiation’ and ‘response to ischemia’) (Figure 6B), while those in an accessible state in the suckling stage were mainly related to immune (e.g., ‘T cell selection’) and metabolic processes (e.g., ‘lipid translocation’ and ‘glycolipid transport’) (Figure 6C), and those accessible in the adult stage were mostly enriched in metabolism (Figure 6D), highlighting the association of ACRs with nutrient and energy homeostasis in the adult porcine liver.

## 3. Discussion

We profiled chromatin accessibility in six organ tissues, including the heart, kidney, liver, lung, skeletal muscle, and spleen, of newborn pigs and explored the associations between tissue-specific ACRs and tissue functions at birth through a combined analysis of ATAC-seq and transcriptome data. We also characterized the chromatin accessibility dynamics in the liver tissues during three developmental stages (i.e., newborn, suckling, and sexually mature stages) and investigated the potential roles of ACRs in the transition of hepatic functions during development, from hematopoiesis to immunity and metabolism, in conjunction with transcriptomic analysis. 

In our study, we noted that the ATAC-seq signals were moderately associated with the gene expression levels. Of note, chromatin open states were also compromised due to DNA replication through disassociate nucleosomes, RNA polymerase, and regulatory factors from DNA [55,56]. Moreover, gene expression was also affected by the other epigenetic factors, such as histone modification and DNA methylation.

Unlike previous studies focusing on the chromatin accessibility of embryonic/fetal or grown pigs [16,17,18,19], our work broadens the epigenetic knowledge of somatic tissues in newborn pigs, which have specific functional characteristics (e.g., by implementing both developmental processes and tissue-specific physical activities) and high value in scientific research (i.e., playing important roles in the postnatal growth of pigs). Furthermore, the tissue-specific ACRs showed partial effects on the gene expression. It was important to explore function divergence between the tissue-specific ACRs consistent with the expression levels and those not consistent with the expression levels. Both types of genes exhibited the specialized functions of the respective tissues (Figure S13), albeit that there were some differences in particular terms, which need to be explored further, especially through an investigation of the regulating roles of the other epigenetic marks in mediating expression changes. In addition, using a union set of PCGs, lncRNAs, and TUCPs, we obtained a relatively comprehensive annotation of open chromatin in multiple porcine tissues, providing an informative resource for genetic studies of farm animals.

Interestingly, although both tissue-specific accessible promoters and other non-coding regions facilitated tissue-specific functions and developmental processes, the latter seemed to have a more important role in morphogenic processes in newborn porcine tissues. Similarly, in the liver, stage-specific ACRs in the intronic and intergenic regions also showed strong evidence supporting their regulatory role in hepatic function transition during development, from hematopoiesis and development in the neonatal stage to the immune and metabolic activities in the adult stage. Therefore, our study not only underlines the regulatory importance of both open promoters and other non-coding ACRs in animals, as previously published research observed [57], but also supports the findings of a recently published 3D genome study that showed that enhancers located in non-coding regions participate in the transcriptional regulation of hepatic function transition during development [58]. 

## 4. Materials and Methods

### 4.1. Ethics

Animal maintenance and experimental procedures were approved by the Institution of Animal Care and Use Committee of the College of Animal Science and Technology, Sichuan Agricultural University, Sichuan, China, under permit DKY-2021202057.

### 4.2. Sample Collection

We collected tissues from two healthy, full-sib, female Berkshire × Tibetan hybrid pigs aged 1 day (newborn stage, denoted as newborn), 28 days (the end of the suckling stage, suckling), and 180 days (sexually mature stage, adult), respectively. Specifically, following euthanasia, the heart, kidney, liver, lung, skeletal muscle, and spleen were collected from the two newborn pigs. Liver tissues were also collected from pigs in the suckling and sexually mature stages. All tissue samples (*n* = 16) were immediately snap-frozen in liquid nitrogen for subsequent high-throughput sequencing.

### 4.3. ATAC-Seq Library Preparation and Sequencing

A total of 16 samples were used to construct libraries for ATAC-seq. We performed the improved ATAC-seq protocol, termed Omni-ATAC [14]. The frozen animal tissues were homogenized in a pre-chilled Dounce and centrifuged in a pre-chilled centrifuge. Avoiding pelleted chunks of connective tissue, 400 uL of homogenate of each sample was transferred to a 2 mL Eppendorf tube. Density gradient centrifugation was conducted by mixing the homogenate with 400 uL of 50% Iodixanol solution and then layering 600 uL of 29% Iodixanol solution and 600 uL of 35% Iodixanol solution under the mixture. The nuclei band was collected, and 50,000 nuclei were transferred into a tube containing 1 mL of ATAC-RSB and 0.1% Tween-20. After centrifugation, the supernatant was discarded, and Omni-ATAC ATAC-seq reaction mix was added to the pellet. The mixture was resuspended and incubated at 37 °C for 30 min for transposition reaction. After a limited PCR cycle to amplify the fragmented DNA, the libraries were purified with AMPure beads. The library quality was assessed with Qubit. The clustering of the index-coded samples was performed with a cBot Cluster Generation System using TruSeq PE Cluster Kit v3-cBot-HS (Illumina, San Diego, CA, USA) following the manufacturer’s instructions. The ATAC-seq libraries were sequenced (150 bp paired-end reads) on a NovaSeq 6000 platform.

### 4.4. ATAC-Seq Data Processing

The adapters and low-quality reads were removed from the raw ATAC-seq data. FastQC was used to examine the quality of the clean reads and high-quality filtered reads aligned with the reference pig genome (Sscrofa 11.1) using Bowtie2 [59] (v 2.2.6) with the following parameters: “-p 8 –end-to-end –very-sensitive –no-mixed –no-discordant –phred33 -t -I 10 -X 700 -q”. Mitochondrial alignments, low-quality alignments (q < 10), and PCR duplicates were removed from the pool of mapped reads using SAMtools [60] (v 1.3.1). The sam files were then converted to .bam files and sorted with SAMtools. The insert size of each sample was calculated using the respective .bam file. We normalized the read counts to a 1× depth (reads per genome coverage, RPGC) for the downstream analyses using the bamCoverage function of deepTools [61] with the following parameters: “--binSize 20000 --normalizeUsing RPGC --extendReads”. Spearman’s correlation coefficients between samples were calculated in 20 kb windows. The ATAC peaks were labeled using MACS2 [62] (https://github.com/macs3-project/MACS accessed on 5 January 2023) with the following options: “--nomodel --extsize 200 --shift -100 --nomodel -B --SPMR --format = BEDPE --keep-dup = 1 --qvalue = 0.05”. We evaluated the enrichment of the ATAC peaks in the TSS regions. IGV [63] (v 2.3.91) was used to visualize the normalized ATAC-seq signals in .bigwig files, which were generated with the merged .bam files of replicates using deepTools’s bamCoverage with RPGC normalization.

### 4.5. Identification of Consensus and Tissue- or Stage-Specific ATAC-Seq Peaks

The reproducible peaks between two biological replicates of the same tissue or stage were termed consensus peaks and identified using the Irreproducible Discovery Rate (IDR) method [64] (v2.0.2) with the default parameters. These peaks represent a comprehensive set of ACRs for certain tissues and/or developmental stages. The genomic distribution of the peaks was annotated in regard to genic features using the ChIPseeker R package [65] (v1.30.3). The peaks were located in promoter (within 2.2 kb upstream and 500 bp downstream of the sequence of TSS), first exon, other exon, first intron, other intron, downstream (within 300 bp downstream of the transcriptional end site), and distal intergenic regions. Differential peaks between two tissues or neighboring stages were identified using the consensus peaks with BEDtools2 [66] (v2.25.0). The tissue- or stage-specific peaks were defined as the consensus peaks that exclusively occurred in one tissue or stage. 

### 4.6. rRNA-Depleted RNA-Seq Library Preparation and Sequencing

To explore the transcriptional profiles of the samples, we downloaded the rRNA-depleted RNA-seq data of the aforementioned newborn tissue samples (*n* = 12) from the Genome Sequence Archive (GSA, https://bigd.big.ac.cn/gsa/ accessed on 20 April 2023; accession number: CRR633913, CRR633924, CRR633914, CRR633925, CRR633948, CRR634010, CRR633915, CRR633926, CRR633938, CRR633950, CRR633916, CRR633927). We constructed strand-specific RNA-seq libraries for the liver tissue samples in the suckling and adult stages (*n* = 4) using an rRNA depletion protocol (Ribo-Zero kit, Epicentre, Madison, WI, USA) coupled with the Illumina TruSeq RNA-seq library protocol. All libraries were quantified using the Qubit dsDNA High-Sensitivity Assay Kit (Invitrogen, Carlsbad, CA, USA) and sequenced with 150 bp paired-end reads on the Illumina HiSeq X Ten platform or, alternatively, with 100 bp paired-end reads on the BGISEQ-500 platform.

### 4.7. RNA-seq Data Processing

The raw reads were trimmed to remove the adapters and low-quality base pairs across samples. The high-quality RNA-seq reads were then aligned with the pig reference genome (Sus scrofa 11.1, release 107) using STAR [67] (v 2.6.0c). A comprehensive set of gene annotations was used, including PCGs, lncRNAs [68], and TUCPs [68]. Gene-level expression was estimated as TPM using the Kallisto software [69] (v 0.43.0) with the default parameters. IGV was used to visualize the locations of the genes and the expression data (average values of two replicates in bigwig files converted from .bam files) of the selected genomic regions. For each tissue, we averaged the replicates and then calculated the tissue specificity of gene abundance using the tau score (τ) [70] (we used 0.75 as the cut-off for tissue-specific genes).

### 4.8. Functional Enrichment Analyses

Functional enrichment analyses were performed using Metascape [71] (http://metascape.org accessed on 28 April 2023). Candidate genes were converted to their human orthologs and used as inputs, taking all the protein-coding genes annotated in the reference human genome as the background. GO-BP and KEGG pathway terms with *p* < 0.01 were considered as statistically significant.

To explore the functional implications of the non-coding open chromatins, we performed enrichment analysis for the tissue- or stage-specific peaks located in intronic and intergenic regions using the GREAT software (v 4.0.4) [72] (http://great.stanford.edu/public/html/ accessed on 30 April 2023) with the default parameters. The homologous regions in the human reference genome (GRCh38) of these peaks were used as inputs.

### 4.9. Motif Enrichment Analysis

Motif enrichment analysis was performed for the peaks of interest using the Analysis of Motif Enrichment tool [73] from MEME [74] (v 5.5.1) with the default settings based on the JASPAR database.

## 5. Conclusions

In summary, our study enriched the epigenetic data reservoir of farm animals but also offered novel insights into the epigenetic features and transcriptional regulation processes in the liver, an essential organ serving as a metabolic hub after birth and closely related to the economic traits (e.g., muscle mass and growth rate) of domestic pigs. Our study lays a theoretical foundation for improving these economic traits in the future.

## Data Availability

The ATAC-seq and RNA-seq data newly generated for this study are available in the Sequence Read Archive (SRA) under the BioProject number PRJNA953794 and Gene Expression Omnibus (GEO) under the accession number GSE229522. High-throughput sequencing ChIP-seq data (H3K4me3 and H3K27me3) were used in this study are available the GEO under accession number GSE158430. High-throughput sequencing RNA-seq data were downloaded in this study are available the GSA under accession number CRA009370.

## Supplementary Materials

The supporting information can be downloaded at: https://www.mdpi.com/article/10.3390/ijms241311076/s1.
