# Supplementary material for: Profiling of Chromatin Accessibility in Pigs across Multiple Tissues and Developmental Stages
Source: Int J Mol Sci. 2023 Jul 4;24(13):11076. doi: 10.3390/ijms241311076 (PMC10341957; doi:10.3390/ijms241311076)
Supplement: Supplementary file 1 [file ijms-24-11076-s001.zip › ijms-2410523-supplementary.pdf]

Supplementary information

Supplementary figures

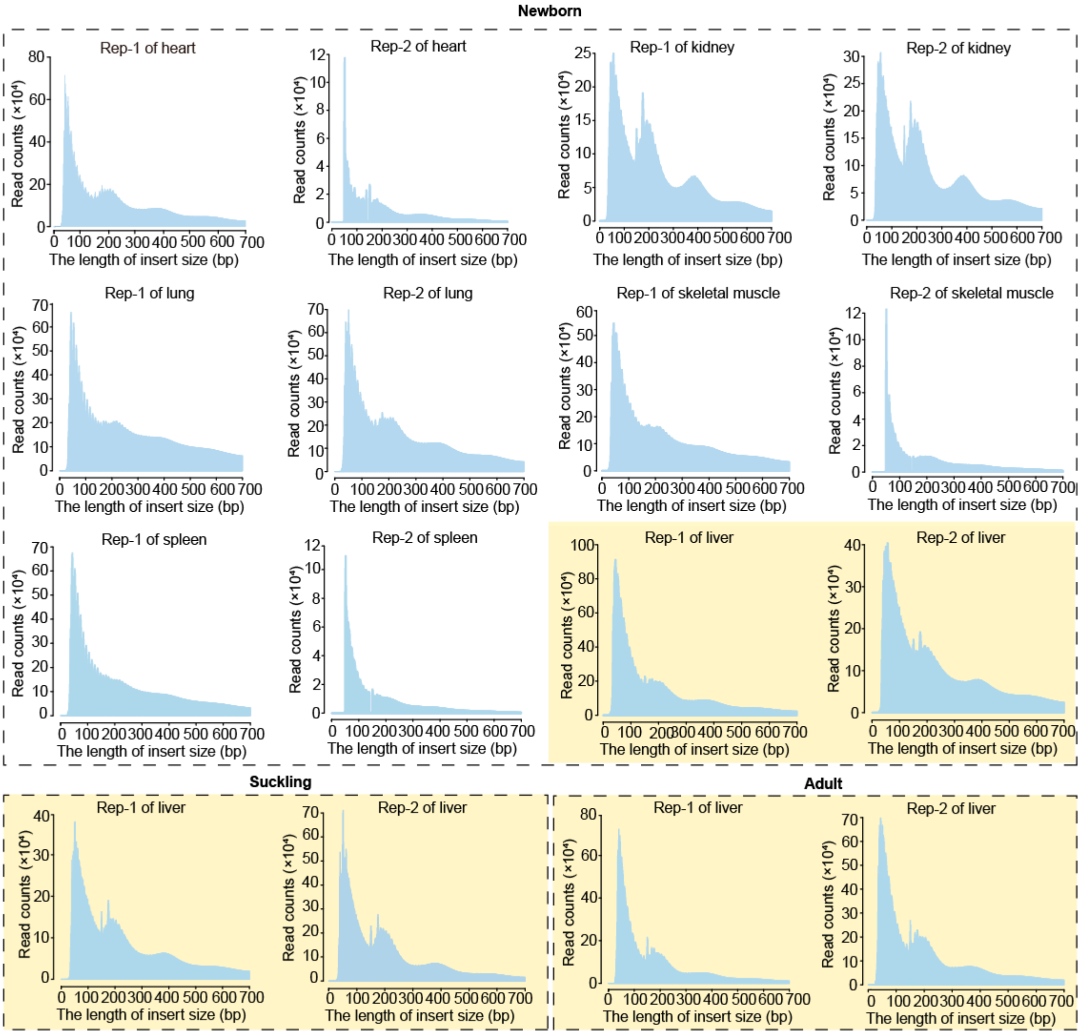

Figure S1. Insert size distribution of all samples (*n* = 16). The yellow squares indicate liver samples across developmental stages (i.e., newborn, suckling, and adult).

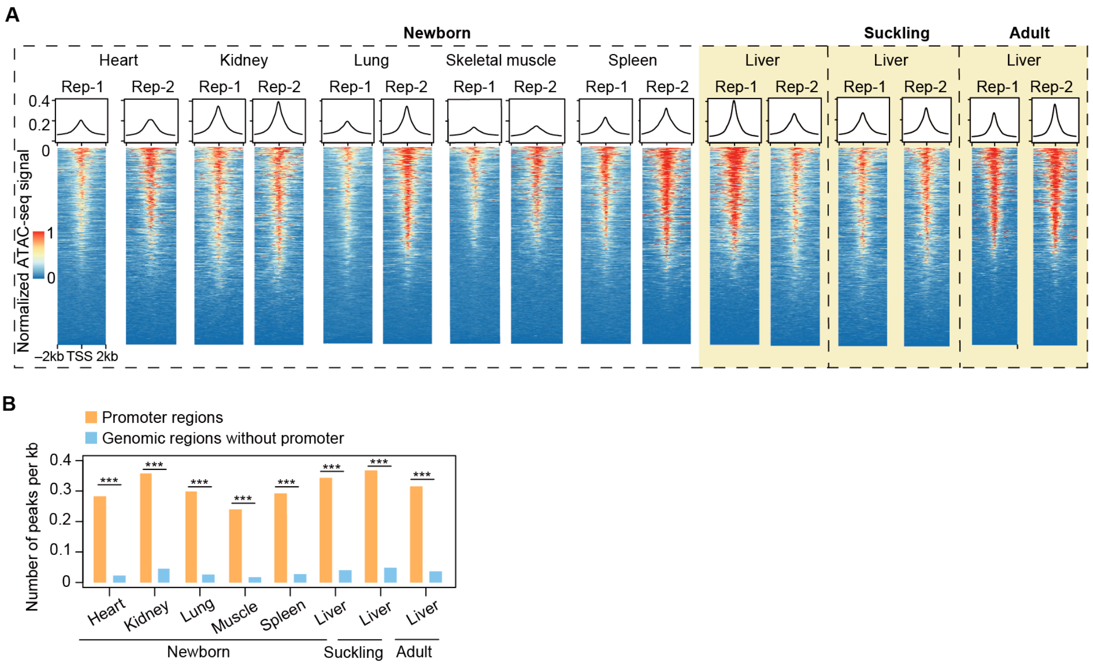

Figure S2. The normalized ATAC-seq signal enriched in TSS and the density of peaks show higher levels in promoter regions than other genomic regions across samples. (A) Line charts (top) and heatmaps (bottom), sorted by normalized ATAC-seq signal intensity, depicting the enrichment of normalized ATAC-seq signal centered on TSS across samples (n = 16). The yellow squares indicate liver samples across developmental stages (i.e., newborn, suckling, and adult). **(B)** The number of peaks per kb are higher in promoter regions (orange) than other genomic regions without promoters (blue; ***$p < 0.001$).

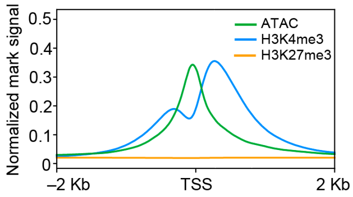

Figure S3. The two types of histone marks (H3K4me3, H3K27me3) for two replicates of liver tissue from pigs at six months were downloaded. The normalized ATAC (green), H3K4me3 (blue) and H3K27me3 (yellow) signal depicting around the TSS of PCGs (n = 22,040).

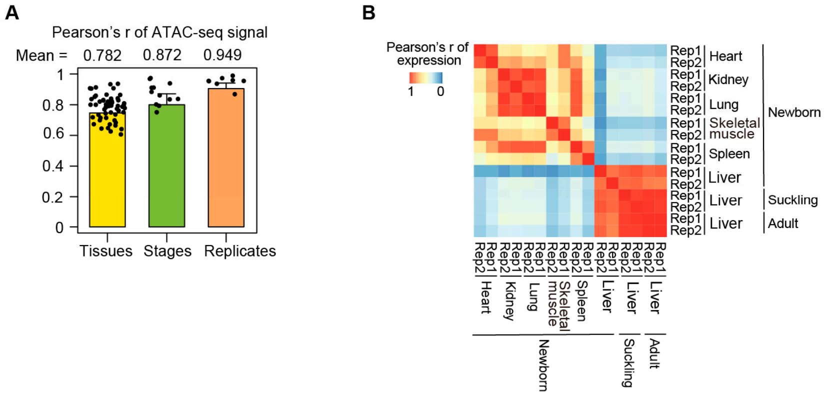

Figure S4. Pearson's correlation of ATAC-seq data and RNA-seq data among the 16 samples. (A) Pearson's correlation coefficients of normalized ATAC-seq signals among 16 samples are shown in three categories (between tissues, between developmental stages of the liver, and between biological replicates). Data are shown as the mean ± SD. **(B)** Pearson's correlation of transcriptional profiles (TPM) among the 16 samples are shown in heatmap.

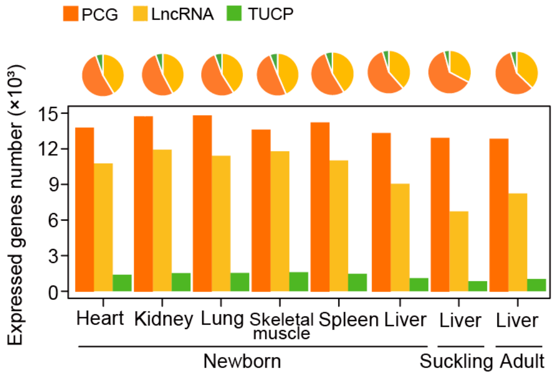

Figure S5. Average percentages (top) and numbers (bottom) of PCGs (TPM > 0.5 in either replicate; orange), lncRNAs (TPM > 0.1 in either replicate; yellow) and TUCPs (TPM > 0.1 in either replicate; green) with detectable expression in each newborn tissue or developmental stage in liver tissue.

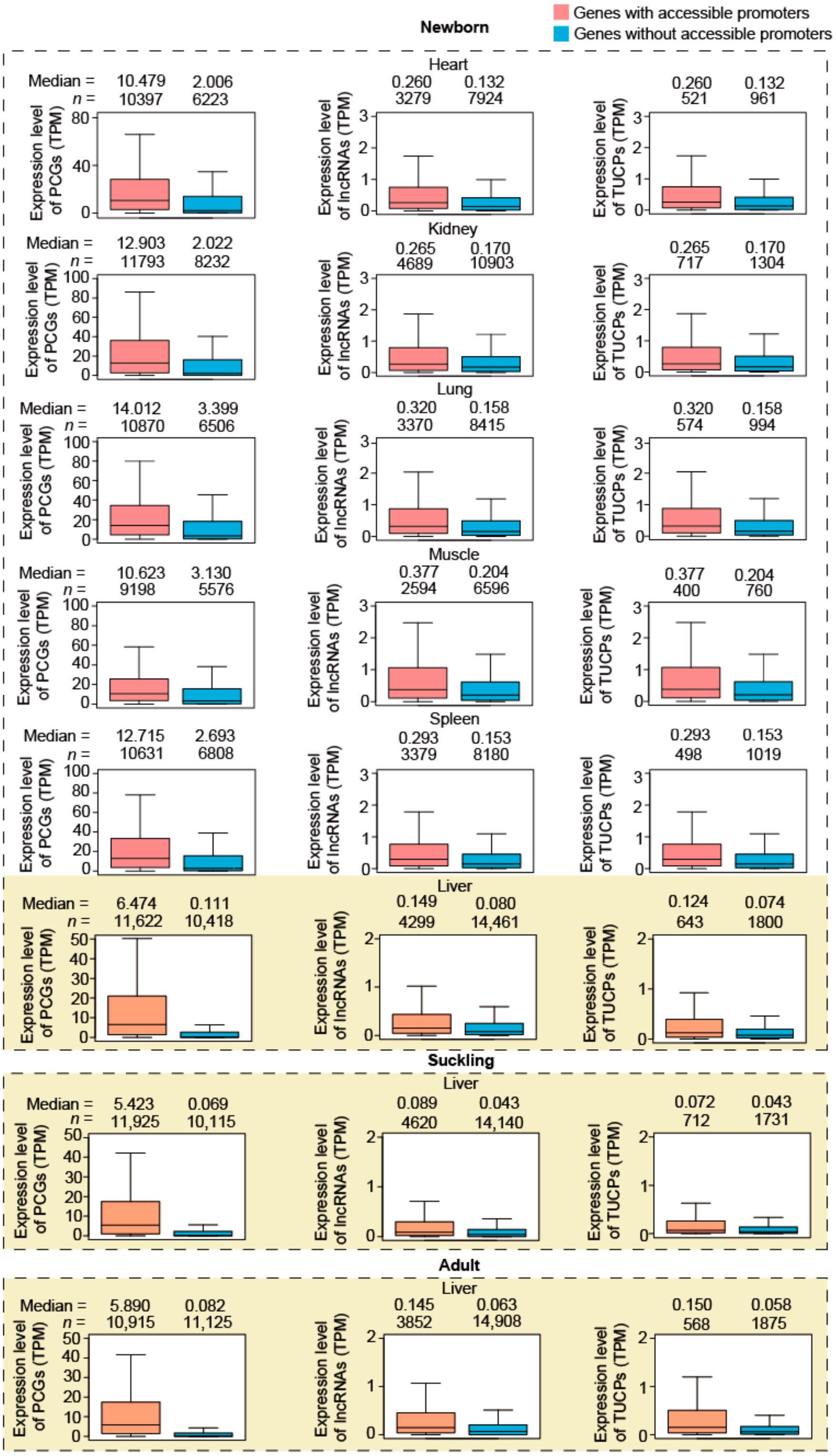

Figure S6. Expression levels (TPM) of PCGs (left), lncRNAs (middle), and TUCPs (right) with accessible promoters showed higher expression than those with no accessible promoters across samples (*n* = 16). Gene numbers are indicated above the plots. Yellow squares indicate liver samples across developmental stages (i.e., newborn, suckling, and adult).

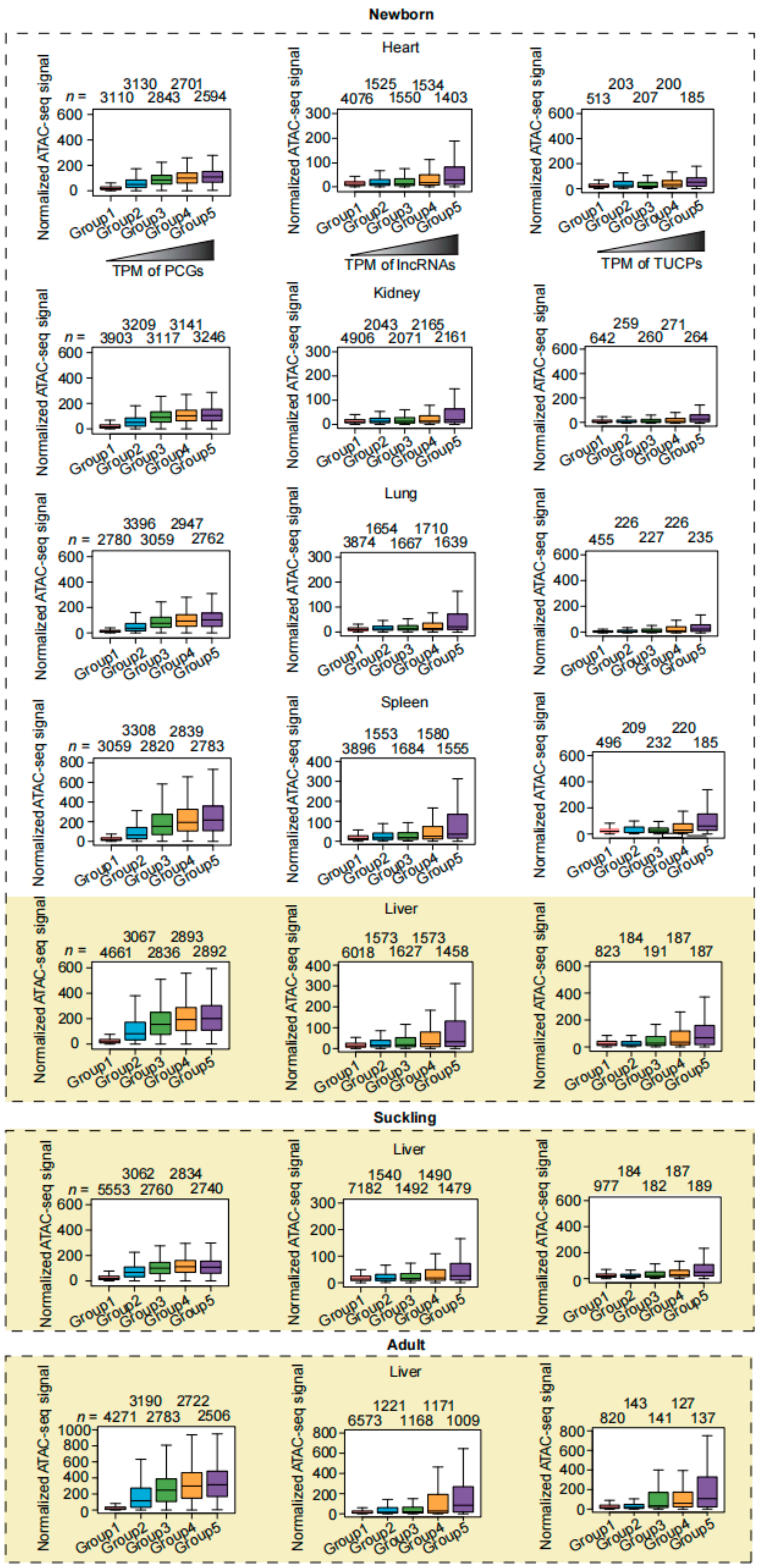

Figure S7. Normalized ATAC-seq signals of PCGs (left), lncRNAs (middle), and TUCPs (right) increased with expression level. Genes were classified into five groups, with expression increasing from group 1 to 5. Gene numbers are indicated above the plots.

The yellow squares indicate liver samples across developmental stages (i.e., newborn, suckling, and adult).

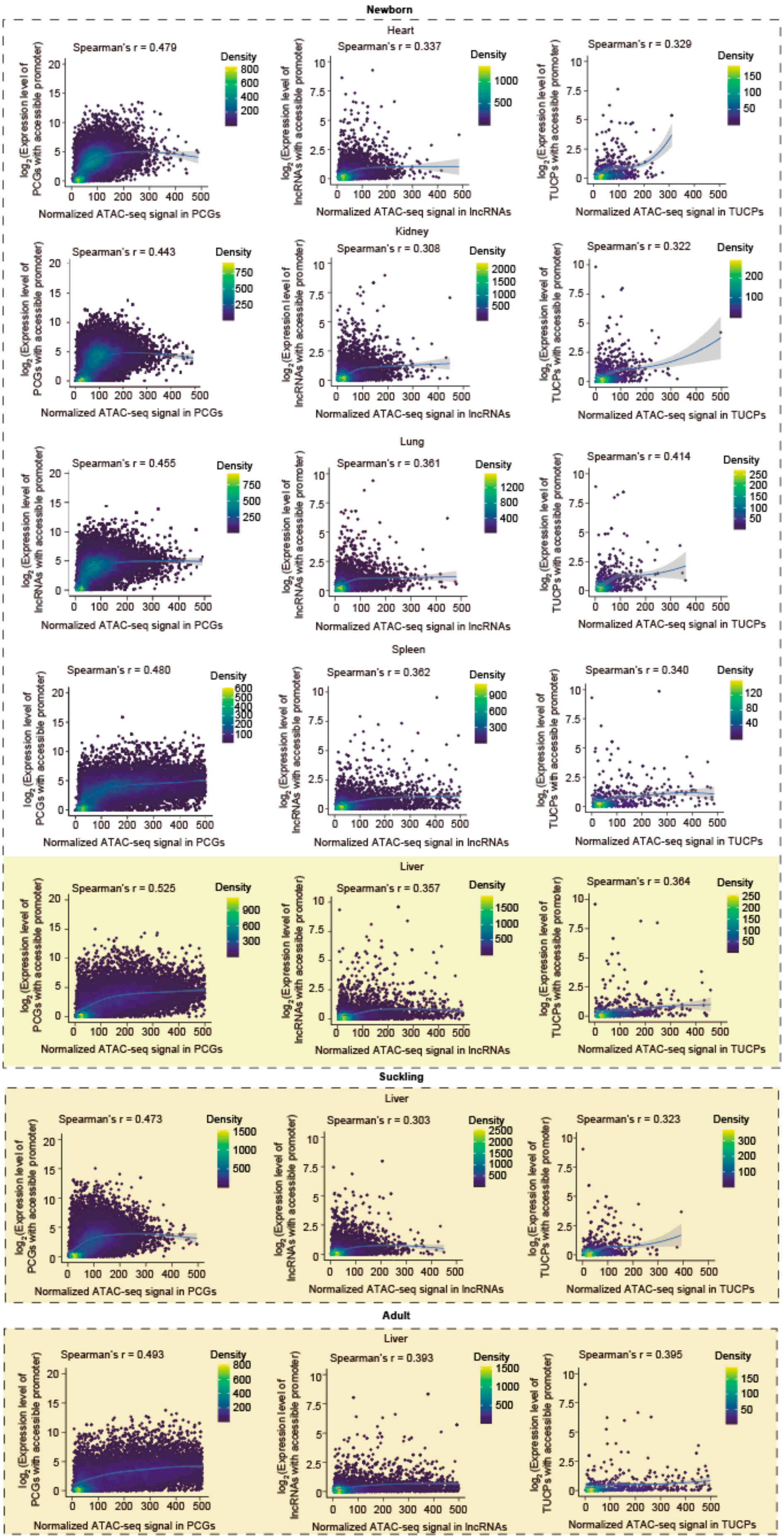

Newborn
Heart
Spearman's r = 0.479
Spearman's r = 0.337
Spearman's r = 0.329
Kidney
Spearman's r = 0.443
Spearman's r = 0.308
Spearman's r = 0.322
Lung
Spearman's r = 0.455
Spearman's r = 0.361
Spearman's r = 0.414
Spleen
Spearman's r = 0.480
Spearman's r = 0.362
Spearman's r = 0.340
Liver
Spearman's r = 0.525
Spearman's r = 0.357
Spearman's r = 0.364
Suckling
Liver
Spearman's r = 0.473
Spearman's r = 0.303
Spearman's r = 0.323
Adult
Liver
Spearman's r = 0.493
Spearman's r = 0.393
Spearman's r = 0.395
Density
log2(Expression level of PCGs with accessible promoter)
log2(Expression level of lncRNAs with accessible promoter)
log2(Expression level of TUCPs with accessible promoter)
Normalized ATAC-seq signal in PCGs
Normalized ATAC-seq signal in lncRNAs
Normalized ATAC-seq signal in TUCPs

Figure S8. The correlation between chromatin accessibility (measured by normalized ATAC-seq signal) and gene expression (measured by TPM using RNA-seq data) in five newborn and liver tissues at the sucking and adult stages. Three types of transcripts are surveyed here, including PCGs, lncRNAs and TUCPs. The gray dashed lines represent the fitting lines. The yellow squares indicate liver samples across developmental stages (i.e., newborn, suckling, and adult).

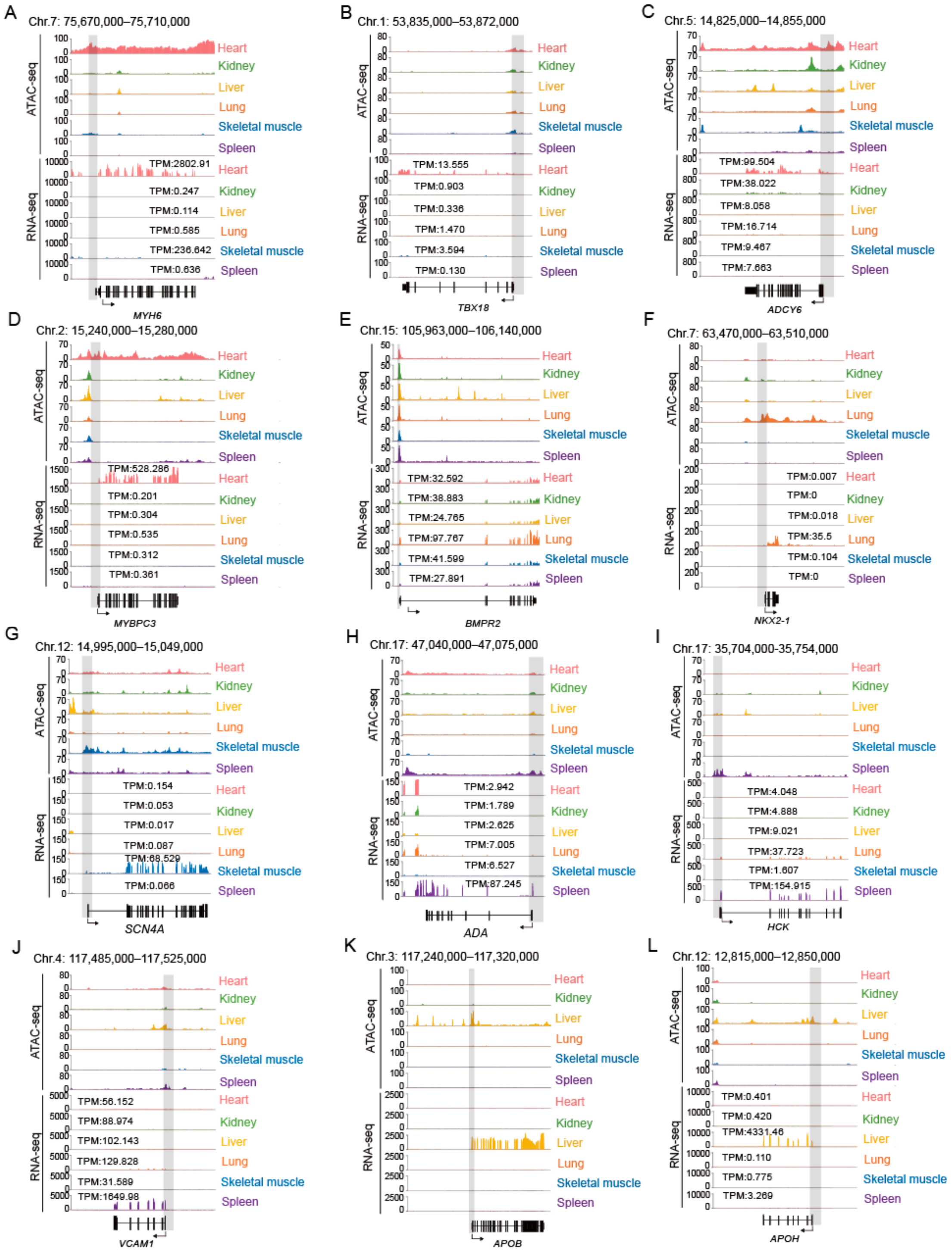

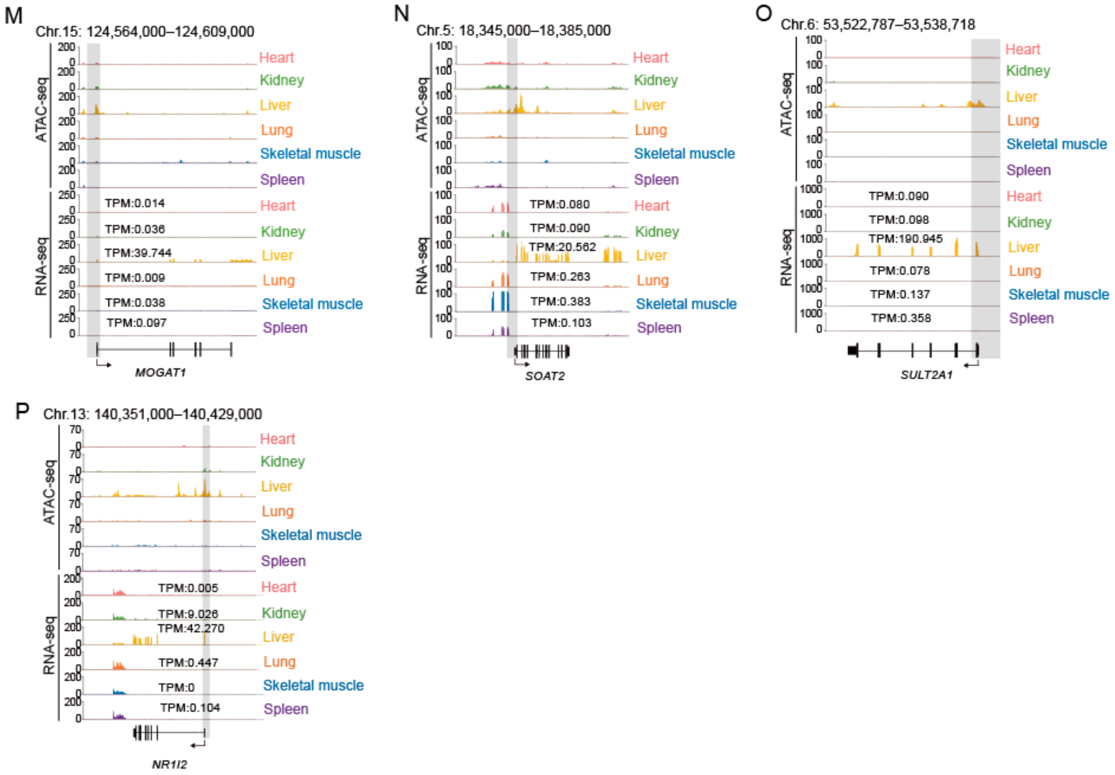

Figure S9. Representative PCGs with tissue-specific accessible promoters and high expression in the corresponding tissue, including *MYH6* (A), *TBX18* (B), *ADCY6* (C), *MYBPC3* (D), *BMPR2* (E), *NKX2-1* (F), *SCN4A* (G), *ADA* (H), *HCK* (I), *VCAM1* (J), *APOB* (K), *APOH* (L), *MOGAT1* (M), *SOAT2* (N), *SULT2A1* (O), and *NR1I2* (P). Chromatin accessibility was measured by the normalized ATAC-seq signals (top tracks), while the expression level was measured by normalized RNA-seq counts (bottom tracks) and TPM (beside the RNA-seq signal tracks). The grey squares indicate the promoter regions of corresponding genes.

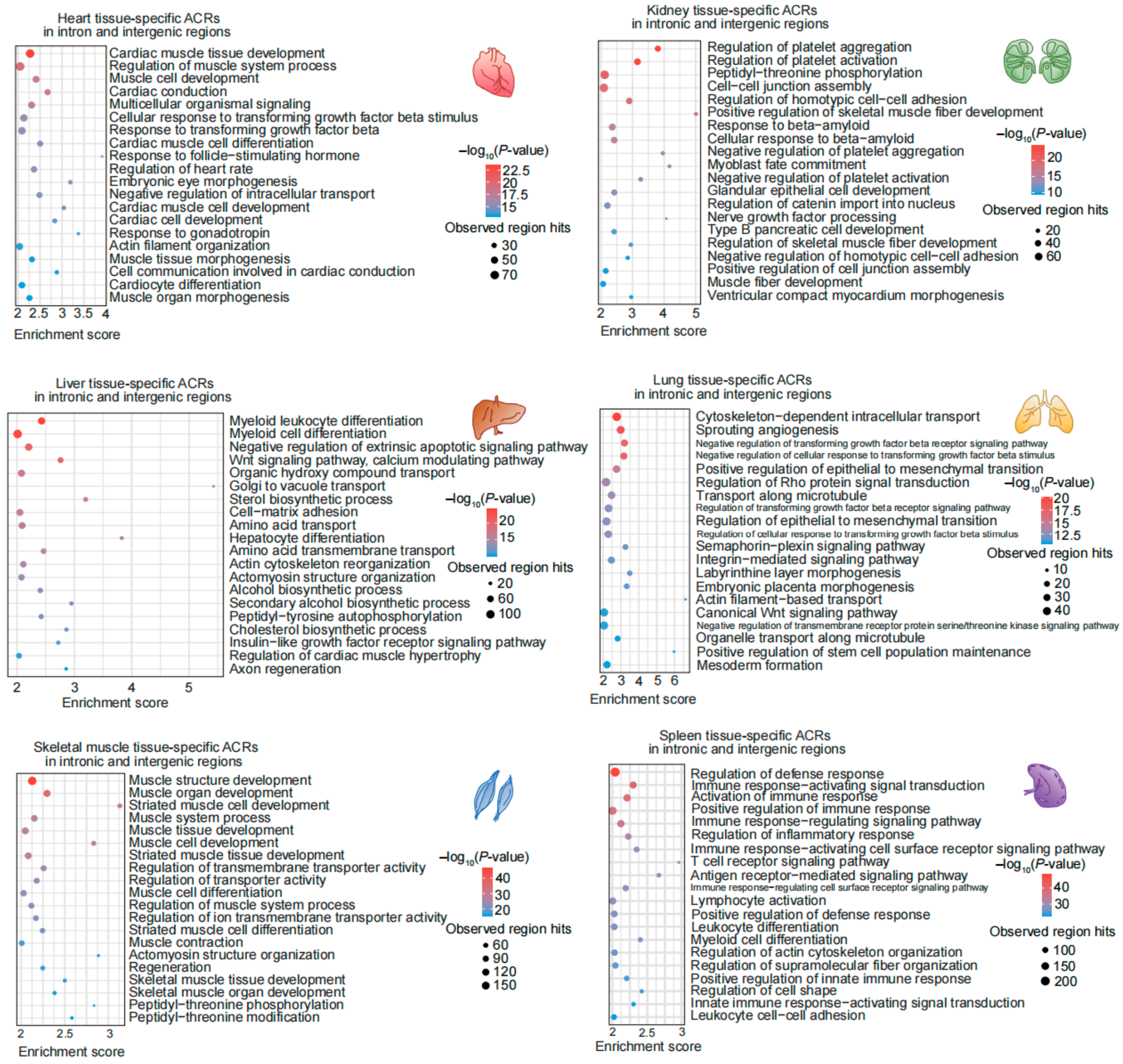

Figure S10. Enrichment of GO-BP terms for tissue-specific ACRs in the intronic and intergenic regions in the heart, kidney, liver, lung, skeletal muscle and spleen tissue at the newborn stage.

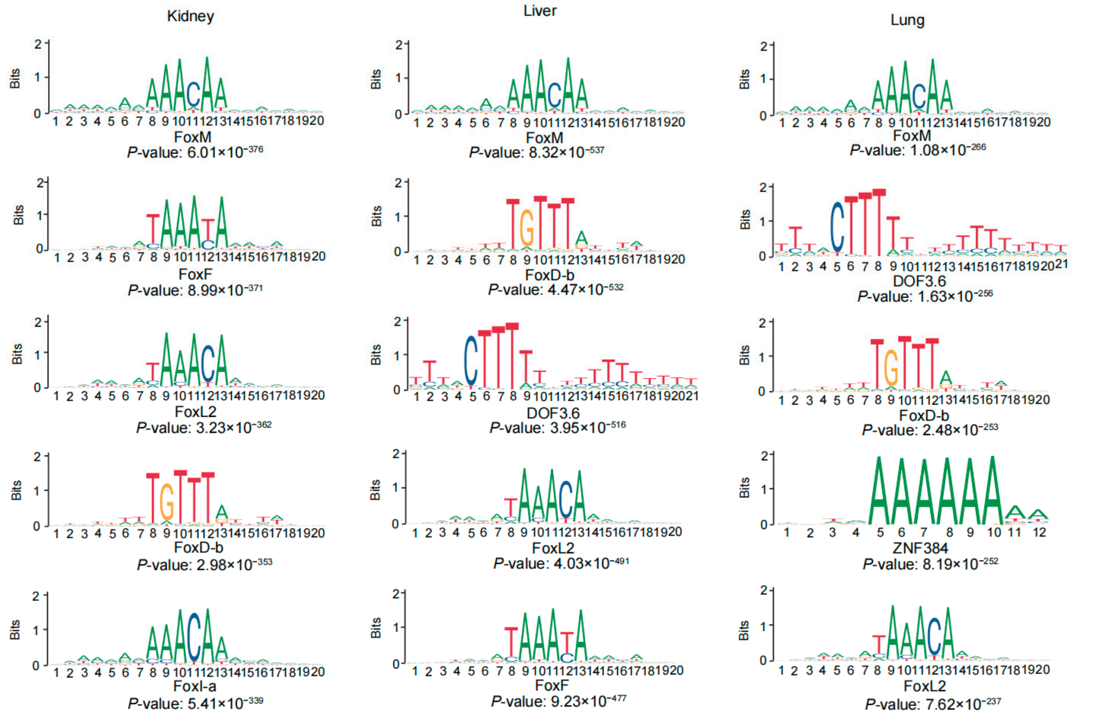

Figure S11. The top five enriched TF binding motifs identified in the kidney- (left), liver- (middle), and lung-specific ACRs (right) using the JASPAR database.

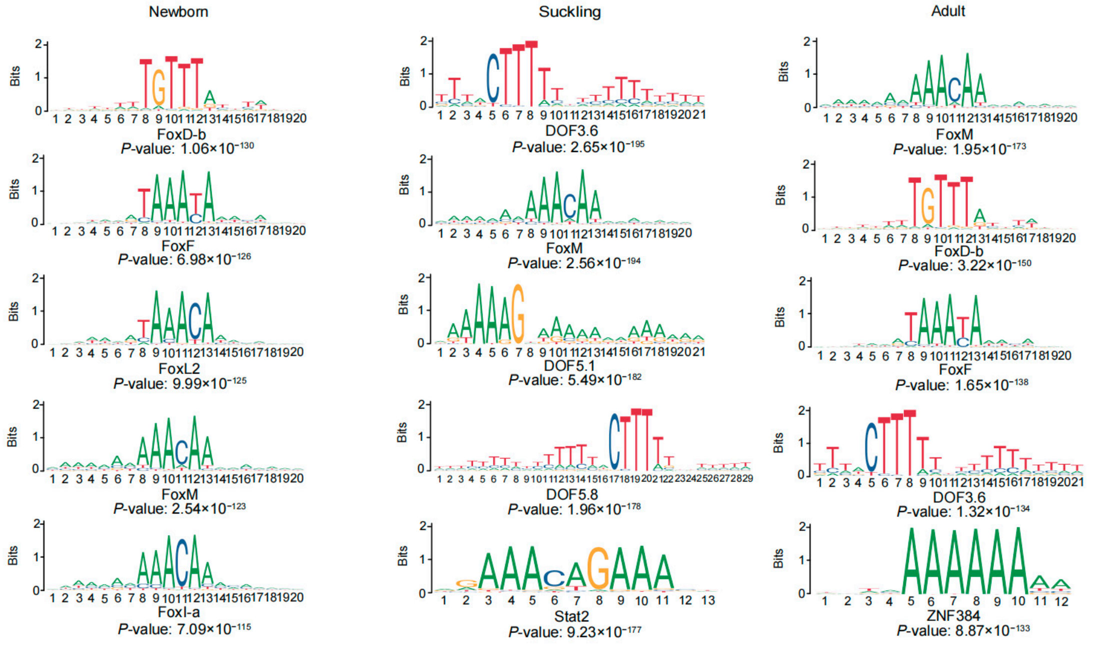

Figure S12. The top five enriched TF binding motifs identified in the newborn (left), sucking (middle), and adult stage-specific ACRs (right) of liver using the JASPAR database.

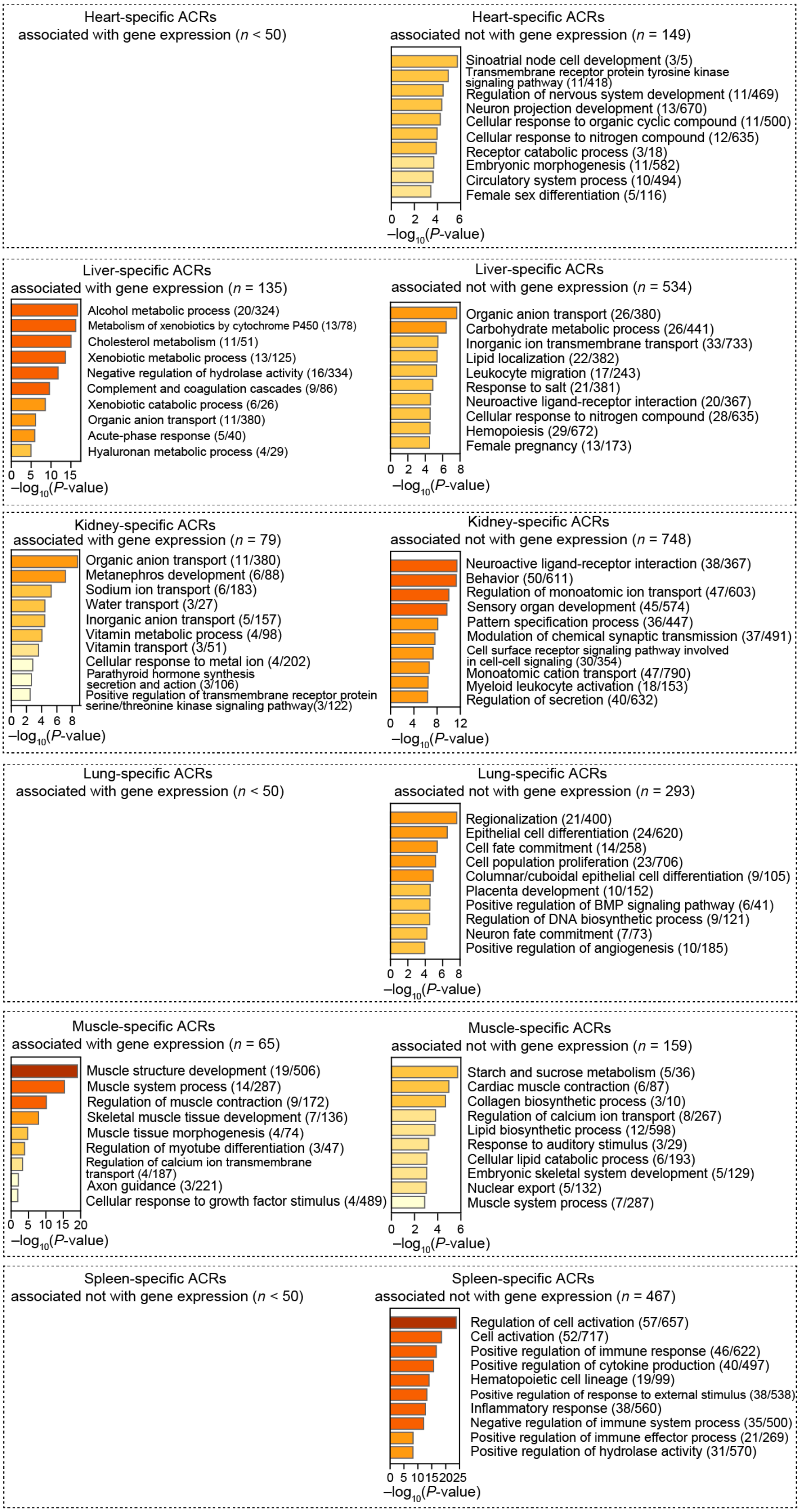

Heart-specific ACRs
associated with gene expression (n < 50)
Heart-specific ACRs
associated not with gene expression (n = 149)
Sinoatrial node cell development (3/5)
Transmembrane receptor protein tyrosine kinase signaling pathway (11/418)
Regulation of nervous system development (11/469)
Neuron projection development (13/670)
Cellular response to organic cyclic compound (11/500)
Cellular response to nitrogen compound (12/635)
Receptor catabolic process (3/18)
Embryonic morphogenesis (11/582)
Circulatory system process (10/494)
Female sex differentiation (5/116)
0 2 4 6
–log10(P-value)
Liver-specific ACRs
associated with gene expression (n = 135)
Alcohol metabolic process (20/324)
Metabolism of xenobiotics by cytochrome P450 (13/78)
Cholesterol metabolism (11/51)
Xenobiotic metabolic process (13/125)
Negative regulation of hydrolase activity (16/334)
Complement and coagulation cascades (9/86)
Xenobiotic catabolic process (6/26)
Organic anion transport (11/380)
Acute-phase response (5/40)
Hyaluronan metabolic process (4/29)
0 5 10 15
–log10(P-value)
Liver-specific ACRs
associated not with gene expression (n = 534)
Organic anion transport (26/380)
Carbohydrate metabolic process (26/441)
Inorganic ion transmembrane transport (33/733)
Lipid localization (22/382)
Leukocyte migration (17/243)
Response to salt (21/381)
Neuroactive ligand-receptor interaction (20/367)
Cellular response to nitrogen compound (28/635)
Hemopoiesis (29/672)
Female pregnancy (13/173)
0 2 4 6 8
–log10(P-value)
Kidney-specific ACRs
associated with gene expression (n = 79)
Organic anion transport (11/380)
Metanephros development (6/88)
Sodium ion transport (6/183)
Water transport (3/27)
Inorganic anion transport (5/157)
Vitamin metabolic process (4/98)
Vitamin transport (3/51)
Cellular response to metal ion (4/202)
Parathyroid hormone synthesis secretion and action (3/106)
Positive regulation of transmembrane receptor protein serine/threonine kinase signaling pathway(3/122)
0 2 4 6 8
–log10(P-value)
Kidney-specific ACRs
associated not with gene expression (n = 748)
Neuroactive ligand-receptor interaction (38/367)
Behavior (50/611)
Regulation of monoatomic ion transport (47/603)
Sensory organ development (45/574)
Pattern specification process (36/447)
Modulation of chemical synaptic transmission (37/491)
Cell surface receptor signaling pathway involved in cell-cell signaling (30/354)
Monoatomic cation transport (47/790)
Myeloid leukocyte activation (18/153)
Regulation of secretion (40/632)
0 4 8 12
–log10(P-value)
Lung-specific ACRs
associated with gene expression (n < 50)
Lung-specific ACRs
associated not with gene expression (n = 293)
Regionalization (21/400)
Epithelial cell differentiation (24/620)
Cell fate commitment (14/258)
Cell population proliferation (23/706)
Columnar/cuboidal epithelial cell differentiation (9/105)
Placenta development (10/152)
Positive regulation of BMP signaling pathway (6/41)
Regulation of DNA biosynthetic process (9/121)
Neuron fate commitment (7/73)
Positive regulation of angiogenesis (10/185)
0 2 4 6 8
–log10(P-value)
Muscle-specific ACRs
associated with gene expression (n = 65)
Muscle structure development (19/506)
Muscle system process (14/287)
Regulation of muscle contraction (9/172)
Skeletal muscle tissue development (7/136)
Muscle tissue morphogenesis (4/74)
Regulation of myotube differentiation (3/47)
Regulation of calcium ion transmembrane transport (4/187)
Axon guidance (3/221)
Cellular response to growth factor stimulus (4/489)
0 5 10 15 20
–log10(P-value)
Muscle-specific ACRs
associated not with gene expression (n = 159)
Starch and sucrose metabolism (5/36)
Cardiac muscle contraction (6/87)
Collagen biosynthetic process (3/10)
Regulation of calcium ion transport (8/267)
Lipid biosynthetic process (12/598)
Response to auditory stimulus (3/29)
Cellular lipid catabolic process (6/193)
Embryonic skeletal system development (5/129)
Nuclear export (5/132)
Muscle system process (7/287)
0 2 4 6
–log10(P-value)
Spleen-specific ACRs
associated not with gene expression (n < 50)
Spleen-specific ACRs
associated not with gene expression (n = 467)
Regulation of cell activation (57/657)
Cell activation (52/717)
Positive regulation of immune response (46/622)
Positive regulation of cytokine production (40/497)
Hematopoietic cell lineage (19/99)
Positive regulation of response to external stimulus (38/538)
Inflammatory response (38/560)
Negative regulation of immune system process (35/500)
Positive regulation of immune effector process (21/269)
Positive regulation of hydrolase activity (31/570)
0 5 10152025
–log10(P-value)

Figure S13. Top ten enriched terms for the tissue-specific ACRs to PCGs consistent or not with expression levels of tissue-specific gene in heart, kidney, liver, lung, skeletal muscle and spleen in newborn stage. Only the enriched terms for tissues with >50 tissue-specific PCGs with specific ACRs are shown. Gene enrichment analysis was performed using the software Metascape.

Supplementary tables

Table S1. Summary of the ATAC-seq data (*n* = 16) generated in this study.

Tissue	Stages	Biological replicates	High-quality reads	Mapped reads	Mapping ratio (%)	Informative reads
Heart	Newborn	Rep1	130,710,817	95,921,818	73.38	76,957,416
		Rep2	82,165,174	68,562,123	83.44	47,472,158
Kidney	Newborn	Rep1	116,082,623	95,991,269	82.69	45,825,946
		Rep2	133,222,601	106,621,134	80.03	56,111,170
Lung	Newborn	Rep1	162,983,352	127,507,349	78.23	106,006,361
		Rep2	157,156,878	127,539,132	81.15	101,964,915
Skeletal muscle	Newborn	Rep1	128,644,239	96,597,915	75.09	79,274,393
		Rep2	84,779,458	67,044,168	79.08	51,384,531
Spleen	Newborn	Rep1	122,841,002	98,587,856	80.26	81,988,801
		Rep2	82,447,044	70,805,623	85.88	53,752,771
Liver	Newborn	Rep1	161,719,547	136,819,053	84.60	93,913,184
		Rep2	142,651,033	110,285,853	77.31	67,630,830
Liver	Suckling	Rep1	137,358,377	100,317,502	73.03	55,182,715
		Rep2	167,117,020	122,646,712	73.39	72,714,120
Liver	Adult	Rep1	157,243,178	94,633,060	60.18	61,540,059
		Rep2	138,886,466	109,345,100	78.73	79,807,245

Table S2. Detailed information of ATAC-seq peaks, consensus peaks and tissue- or stage-specific peaks identified in the samples (*n* = 16).

Tissue	Stages	Biological replicates	Peaks numbers	Consensus peak numbers	No. of consensus peaks overlapping with			No. of tissue- or stage-specific peaks overlapping with			No. of genes or transcripts overlapping with tissue- or stage-specific peaks			No. of PCGs with promoters overlapping with peaks
					PCG	lncRNA	TUCP	PCG	lncRNA	TUCP	PCG	lncRNA	TUCP	
Heart	Newborn	Rep1	160,462	72920	38841	28044	3505	5565	6139	679	3490	3809	422	220
		Rep2	141,951											
Kidney	Newborn	Rep1	221,461	133254	66502	54701	6980	19769	21998	2702	8649	8800	1067	970
		Rep2	244,304											
Lung	Newborn	Rep1	183,598	80657	43335	30307	3765	8059	8068	839	4792	4779	531	410
		Rep2	205,830											
Skeletal muscle	Newborn	Rep1	115,523	57126	30846	21334	2649	5642	5939	637	3442	3538	384	270
		Rep2	118,584											
Spleen	Newborn	Rep1	173,694	84327	45934	31524	3830	9982	8932	926	5578	5029	571	574
		Rep2	149,660											
Liver	Newborn	Rep1	240,651	120012	64853	45111	5539	17011	13839	1565	8183	6867	823	824
		Rep2	270,293					10545	9043	1061	6480	5622	668	731
Liver	Suckling	Rep1	275,336	141689	75237	53522	6875	17823	15910	2199	8826	4823	1020	1028
		Rep2	286,446											
Liver	Adult	Rep1	136,452	109003	59707	40357	4705	10611	8444	851	5875	7734	513	612
		Rep2	190,255											

Table S3. Summary of the RNA-seq data (*n* = 16) used in this study, including 12 newborn porcine tissue samples downloaded from the GSA database.

Tissue	Stages	Biological replicates	High-quality data (Gb)	Uniquely mapped data (Gb)	Uniquely mapped ratio	Expressed genes number		
						PCG	lncRNA	TUCP
Heart	Newborn	Rep1	15.98	13.35	83.57	13827	10764	1397
		Rep2	16.13	13.85	85.82			
Kidney	Newborn	Rep1	16.03	13.74	85.7	14777	11921	1533
		Rep2	15.47	13.89	89.8			
Lung	Newborn	Rep1	15.98	12.87	80.55	14860	11421	1543
		Rep2	16.12	13.87	86.03			
Skeletal muscle	Newborn	Rep1	16.54	11.91	71.99	13657	11789	1601
		Rep2	15.61	13.01	83.34			
Spleen	Newborn	Rep1	16.00	13.70	85.64	14267	11025	1470
		Rep2	15.74	13.47	85.53			
Liver	Newborn	Rep1	16.48	13.01	78.91	13372	9061	1109
		Rep2	16.84	12.30	73.06			
Liver	Suckling	Rep1	15.23	12.29	80.71	12966	6732	847
		Rep2	15.46	12.34	79.79			
Liver	Adult	Rep1	15.43	11.16	72.3	12891	8236	1022
		Rep2	15.45	12.04	77.93			

Table S4. Detailed information of the top ten TF binding motifs identified in the tissue-specific ATAC-seq peaks.

Tissue	Rank	Motif ID*	Alternate ID*	Consensus*	*P*-value*	E-value*
Heart	1	MA0052.4	MEF2A	DDCTAAAAATAGMHH	1.12×10^{-199}	6.41×10^{-193}
	2	MA0497.1	MEF2C	DDDCYAAAAATAGMW	4.43×10^{-193}	2.11×10^{-186}
	3	MA0940.1	AP1	MYAAAAAWRGAAA	1.39×10^{-112}	1.00×10^{-105}
	4	MA1623.1	Stat2	RGAAACAGAAASH	1.32×10^{-111}	8.37×10^{-105}
	5	MA0660.1	MEF2B	RCTAWAAATAGC	1.56×10^{-109}	1.79×10^{-103}
	6	MA0773.1	MEF2D	DCTAWAAATAGM	8.99×10^{-103}	1.01×10^{-96}
	7	MA1274.1	DOF3.6	TTTWCTTTTTHHYTTTTTTTT	2.35×10^{-99}	1.24×10^{-92}
	8	MA1823.1	Zm00001d027846	RRAAAGAAAARR	2.77×10^{-94}	2.48×10^{-87}
	9	MA0543.1	eor-1	RRAGAGASRSAGAGA	6.38×10^{-93}	3.54×10^{-86}
	10	MA1871.1	FoxM	HAAAMAHAAACAAHMAHAHH	4.39×10^{-92}	3.16×10^{-85}
Kidney	1	MA0052.4	MEF2A	DDCTAAAAATAGMHH	1.12×10^{-199}	6.41×10^{-193}
	2	MA0497.1	MEF2C	DDDCYAAAAATAGMW	4.43×10^{-193}	2.11×10^{-186}
	3	MA0940.1	AP1	MYAAAAAWRGAAA	1.39×10^{-112}	1×10^{-105}
	4	MA1623.1	Stat2	RGAAACAGAAASH	1.32×10^{-111}	8.37×10^{-105}
	5	MA0660.1	MEF2B	RCTAWAAATAGC	1.56×10^{-109}	1.79×10^{-103}
	6	MA0773.1	MEF2D	DCTAWAAATAGM	8.99×10^{-103}	1.01×10^{-96}
	7	MA1274.1	DOF3.6	TTTWCTTTTTHHYTTTTTTTT	2.35×10^{-99}	1.24×10^{-92}
	8	MA1823.1	Zm00001d027846	RRAAAGAAAARR	2.77×10^{-94}	2.48×10^{-87}
	9	MA0543.1	eor-1	RRAGAGASRSAGAGA	6.38×10^{-93}	3.54×10^{-86}
	10	MA1871.1	FoxM	HAAAMAHAAACAAHMAHAHH	4.39×10^{-92}	3.16×10^{-85}
Liver	1	MA1871.1	FoxM	HAAAMAHAAACAAHMAHAHH	6.51×10^{-541}	1.63×10^{-533}
	2	MA1862.1	FoxD-b	DDDTDWWTGTTTAYDTWDNN	3.43×10^{-536}	8.74×10^{-529}
	3	MA1274.1	DOF3.6	TTTWCTTTTTHHYTTTTTTTT	4.38×10^{-520}	7.72×10^{-513}
	4	MA1870.1	FoxL2	NHWWAHATAAACAAWHMHHH	3.28×10^{-495}	7.88×10^{-488}

	5	MA1864.1	FoxF	NNNHWHATAAATAWWHANHN	9.07×10^{-481}	1.81×10^{-473}
	6	MA1869.1	FoxK	NDDDDDDTGTTTAYDDDNNN	6.63×10^{-472}	1.84×10^{-464}
	7	MA1281.1	DOF5.1	RAAAAAGWAAAAARAAAAARA	2.15×10^{-456}	3.67×10^{-449}
	8	MA1267.1	DOF5.8	WHTTTTTTHYTTTTTACTTTTTNHTTTWW	6.11×10^{-450}	9.70×10^{-443}
	9	MA1866.1	FoxI-a	HWAAHAHAAACAAAMMHHHN	2.17×10^{-443}	5.62×10^{-436}
	10	MA1125.1	ZNF384	DNWMAAAAAAAA	2.69×10^{-432}	4.51×10^{-425}
Lung	1	MA1871.1	FoxM	HAAAMAHAAACAAHMAHAHH	1.67×10^{-270}	2.12×10^{-263}
	2	MA1274.1	DOF3.6	TTTWCTTTTTHHYTTTTTTTT	3.37×10^{-260}	3.19×10^{-253}
	3	MA1862.1	FoxD-b	DDDTDWWTGTTTAYDTWDNN	3.74×10^{-257}	4.85×10^{-250}
	4	MA1125.1	ZNF384	DNWMAAAAAAAA	1.74×10^{-255}	1.6×10^{-248}
	5	MA1870.1	FoxL2	NHWWAHATAAACAAWHMHHH	1.22×10^{-240}	1.49×10^{-233}
	6	MA1864.1	FoxF	NNNHWHATAAATAWWHANHN	5.24×10^{-226}	5.28×10^{-219}
	7	MA1869.1	FoxK	NDDDDDDTGTTTAYDDDNNN	6.78×10^{-220}	9.41×10^{-213}
	8	MA1267.1	DOF5.8	WHTTTTTTHYTTTTTACTTTTTNHTTTWW	4.39×10^{-212}	3.7×10^{-205}
	9	MA1866.1	FoxI-a	HWAAHAHAAACAAAMMHHHN	2.34×10^{-205}	2.98×10^{-198}
	10	MA1281.1	DOF5.1	RAAAAAGWAAAAARAAAAARA	8.78×10^{-201}	7.93×10^{-194}
Skeletal muscle	1	MA0052.4	MEF2A	DDCTAAAAATAGMHH	1.32×10^{-170}	7.42×10^{-164}
	2	MA0497.1	MEF2C	DDDCYAAAAATAGMW	1.79×10^{-155}	8.23×10^{-149}
	3	MA1845.1	Atoh7	NNNNRRCAGCTGTYNNNNNN	8.17×10^{-154}	8.35×10^{-147}
	4	MA1100.2	ASCL1	VGCAGCTGCN	1.45×10^{-148}	1.63×10^{-141}
	5	MA0499.2	MYOD1	NNGCACCTGTCNB	4.22×10^{-141}	3.51×10^{-134}
	6	MA0816.1	Ascl2	ARCAGCTGCY	5.29×10^{-135}	3.83×10^{-128}
	7	MA1641.1	MYF5	NVACAGCTGTBN	4×10^{-134}	3.01×10^{-127}
	8	MA1472.2	Bhlha15	NVACAGCTGTBN	5.99×10^{-133}	4.45×10^{-126}
	9	MA1619.1	Ptf1A	NNACAGCTGTNN	7.55×10^{-129}	5.96×10^{-122}
	10	MA0521.2	Tcf12	NNACAGCTGTNN	1.63×10^{-127}	1.23×10^{-120}

Spleen	1	MA1853.1	Erf-a	NNNNRNCGGAAGTNNNNNNN	3.76×10^{-500}	5.94×10^{-493}
	2	MA0080.6	Spi1	RRAAAGAGGAAGTGGDD	6.47×10^{-484}	8.77×10^{-477}
	3	MA0598.3	EHF	NNCACTTCCTGTTNN	5.35×10^{-474}	7.71×10^{-467}
	4	MA0081.2	SPIB	TYTCACTTCCTCTTTY	3.55×10^{-425}	4.37×10^{-418}
	5	MA0474.3	Erg	NNACAGGAAGTGVN	4.58×10^{-425}	6.55×10^{-418}
	6	MA0640.2	ELF3	NNCCACTTCCTGNT	1.77×10^{-412}	2.79×10^{-405}
	7	MA0761.2	ETV1	NNACAGGAAGTGNN	2.77×10^{-391}	3.94×10^{-384}
	8	MA0062.3	GABPA	NNCACTTCCTGTNN	5.15×10^{-389}	7.63×10^{-382}
	9	MA0136.3	Elf5	RVAAGGAAGTNN	9.89×10^{-369}	1.66×10^{-361}
	10	MA1508.1	IKZF1	VVAACAGGAARN	3.56×10^{-365}	6.18×10^{-358}

*Motif ID: The name of the motif, which is unique in the JASPAR database.

*Alternate ID: An alternate name for the motif that may be provided in the JASPAR database.

*Consensus: A consensus sequence computed from the motif.

**P*-value: The optimal enrichment p-value of the motif according to the statistical test, adjusted for multiple tests using a Bonferroni correction.

*E-value: The expected number of random motifs that would be as enriched in the (primary) sequences as this one. The E-value is the adjusted p-value multiplied by the number of motifs in the motif file.

Table S5. Detailed information of the top ten TF binding motifs identified in the stage-specific ATAC-seq peaks.

Tissue	Rank	Motif ID	Alternate ID	Consensus	*P*-value	E-value
Newborn	1	MA1862.1	FoxD-b	DDDTDWWTGTTTAYDTWDNN	2.91×10^{-134}	2.07×10^{-127}
	2	MA1864.1	FoxF	NNNHWHATAAATAWWHANHN	2.41×10^{-129}	1.37×10^{-122}
	3	MA1870.1	FoxL2	NHWWAHATAAACAAWHMHHH	2.85×10^{-128}	1.95×10^{-121}
	4	MA1871.1	FoxM	HAAAMAHAAACAAHMAHAHH	7.25×10^{-127}	4.96×10^{-120}
	5	MA1866.1	FoxI-a	HWAAHAHAAACAAAMMHHHN	1.92×10^{-118}	1.39×10^{-111}
	6	MA1869.1	FoxK	NDDDDDDTGTTTAYDDDNNN	8.35×10^{-116}	6.71×10^{-109}
	7	MA1274.1	DOF3.6	TTTWCTTTTTHHYTTTTTTTT	5.72×10^{-113}	2.52×10^{-106}
	8	MA1623.1	Stat2	RGAAACAGAAASH	3.21×10^{-105}	1.88×10^{-98}
	9	MA1281.1	DOF5.1	RAAAAAGWAAAAARAAAAARA	5.51×10^{-105}	2.35×10^{-98}
	10	MA1267.1	DOF5.8	WHTTTTTTHYTTTTTACTTTTTNHTTTWW	4.54×10^{-103}	1.74×10^{-96}
Suckling	1	MA1274.1	DOF3.6	TTTWCTTTTTHHYTTTTTTTT	8.68×10^{-199}	5.18×10^{-192}
	2	MA1871.1	FoxM	HAAAMAHAAACAAHMAHAHH	5.72×10^{-198}	5.01×10^{-191}
	3	MA1281.1	DOF5.1	RAAAAAGWAAAAARAAAAARA	1.9×10^{-185}	1.07×10^{-178}
	4	MA1267.1	DOF5.8	WHTTTTTTHYTTTTTACTTTTTNHTTTWW	1.2×10^{-181}	5.79×10^{-175}
	5	MA1623.1	Stat2	RGAAACAGAAASH	2.1×10^{-180}	1.81×10^{-173}
	6	MA0940.1	AP1	MYAAAAAWRGAAA	9.6×10^{-170}	8.16×10^{-163}
	7	MA1125.1	ZNF384	DNWMAAAAAAAA	1.53×10^{-163}	7.48×10^{-157}
	8	MA0002.2	Runx1	BBYTGTGGTTT	8.93×10^{-163}	1.44×10^{-155}
	9	MA1866.1	FoxI-a	HWAAHAHAAACAAAMMHHHN	2.42×10^{-162}	2.35×10^{-155}
	10	MA1823.1	Zm00001d027846	RRAAAGAAAARR	6.76×10^{-161}	7.32×10^{-154}
Adult	1	MA1871.1	FoxM	HAAAMAHAAACAAHMAHAHH	3.83×10^{-177}	3.82×10^{-170}
	2	MA1862.1	FoxD-b	DDDTDWWTGTTTAYDTWDNN	6.46×10^{-154}	6.29×10^{-147}
	3	MA1864.1	FoxF	NNNHWHATAAATAWWHANHN	4.53×10^{-142}	3.24×10^{-135}

4	MA1274.1	DOF3.6	TTTWCTTTTTHHYTTTTTTTT	3.66×10^{-138}	2.58×10^{-131}
5	MA1125.1	ZNF384	DNWMAAAAAAAA	2.44×10^{-136}	1.73×10^{-129}
6	MA1870.1	FoxL2	NHWWAHATAAACAAWHMHHH	2.43×10^{-133}	2.19×10^{-126}
7	MA1866.1	FoxI-a	HWAAHAHAAACAAAMMHHHN	6.13×10^{-125}	6.03×10^{-118}
8	MA1267.1	DOF5.8	WHTTTTTTHYTTTTTACTTTTTNHTTTWW	4.83×10^{-118}	3.10×10^{-111}
9	MA1869.1	FoxK	NDDDDDDTGTTTAYDDDNNN	2.32×10^{-111}	2.39×10^{-104}
10	MA1281.1	DOF5.1	RAAAAAGWAAAAARAAAAARA	$3.82\times10_{-108}$	2.53×10^{-101}
